# Capsid-engineered AAV vector overcomes a key intracellular barrier and efficiently transduces spiral ganglion neurons in adult mice

**DOI:** 10.1016/j.omta.2026.201669

**Published:** 2026-01-13

**Authors:** Jennifer Marx, Peixin Huang, Sereina O. Sutter, Moritz Ertelt, Odett Kaiser, Jennifer Harre, Juliane Schott, Josephine Wilkes, Philipp Neek-John, Axel Rossi, Athanasia Warnecke, Clara T. Schoeder, Axel Schambach, Hinrich Staecker, Hildegard Büning

**Affiliations:** 1 Institute of Experimental Hematology, Hannover Medical School, 30625 Hannover, Germany; 2 Department of Otolaryngology Head and Neck Surgery, University of Kansas School of Medicine, Kansas City, KS 66160, USA; 3 Institute of Virology, University of Zurich, Zurich, Switzerland; 4 Institute for Drug Discovery, Leipzig University Faculty of Medicine, 04103 Leipzig, Germany; 5 Center for Scalable Data Analytics and Artificial Intelligence ScaDS.AI, Dresden/Leipzig, Germany; 6 Department of Otolaryngology, Head and Neck Surgery, Hannover Medical School, 30625 Hannover, Germany; 7 Division of Hematology/Oncology, Boston Children's Hospital; Department of Pediatric Oncology, Dana-Farber Cancer Institute; Harvard Medical School, Boston, MA, USA

**Keywords:** Adeno-associated virus, AAV, vector, gene therapy, inner ear, spiral ganglion neurons, uncoating, BDNF, capsid engineering

## Abstract

Hearing loss, the most prevalent sensory disorder, can result from pathology in various cochlear cell types. To develop effective gene therapy-based treatment strategies, vectors must be adapted to meet cell type-specific requirements. Here, we report a novel adeno-associated virus (AAV) capsid variant, AAV.MPI, which transduces spiral ganglion neurons (SGNs) in mature mouse cochleae with remarkable efficacy, even at low doses. This unique feature is mediated by a heptamer peptide, identified by a phage display peptide library screen, and inserted into the GH12/GH13 loop in each of the 60 subunits forming the mature AAV2 capsid. In addition to its change in vector tropism, AAV.MPI showed higher transgene expression efficacy. In line with this, uncoating assays revealed a more efficient release of vector genomes from AAV.MPI compared to AAV2 capsids, both *in vitro* and *in vivo*. Uncoating of AAV.MPI—in contrast to AAV2—likely occurs independently of cell cycle progression. Exploring AAV.MPI’s SGN-tropism, SGN degeneration in deafened mice was prevented by vector-mediated overexpression of brain-derived neurotrophic factor. In summary, we present AAV.MPI as a novel AAV capsid variant adapted for cochlear gene therapy targeting SGNs. We also highlight vector uncoating as a key limiting post-entry step in auditory cell transduction.

## Graphical abstract

**Figure:**
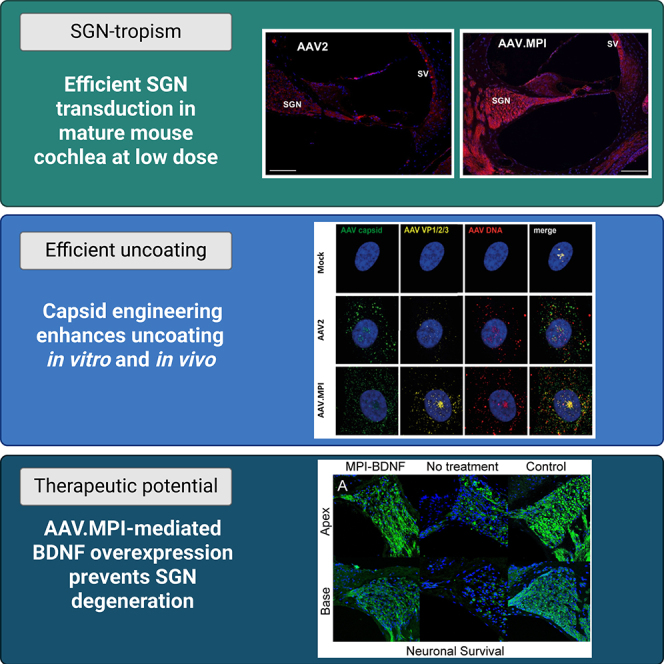

## Teaser

AAV.MPI is a capsid variant that efficiently transduces spiral ganglion neurons across the adult mouse cochlea. This advance enables targeted gene therapy for hearing loss. Moreover, this study identified vector uncoating as a key barrier to inner ear cell transduction, providing new insight into vector design for sensory disorders.

## Introduction

Sensorineural hearing loss (SNHL) is a prevalent sensory impairment affecting approximately 20% of the global human population, with significant implications for communication, social interaction, and overall quality of life.^1^^,^^2^ SNHL is primarily characterized by dysfunction or degeneration of inner ear structures, including hair cells (HCs) and spiral ganglion neurons (SGNs). While current hearing devices, including cochlear implants (CIs), have markedly improved outcomes for many individuals, challenges persist in achieving optimal hearing sensitivity and natural sound perception, particularly in complex acoustic environments.^3^^,^^4^^,^^5^ Moreover, the efficacy of CIs, which bypass damaged HCs to directly stimulate surviving SGNs, varies considerably among recipients due to factors such as surgery-associated damage, SGN survival rates, onset of hearing loss, and individual differences in sound perception.^6^^,^^7^^,^^8^^,^^9^ To address this variability, auxiliary gene therapy approaches are being developed to enhance CI performance and overall auditory function. In this regard, neurotrophic factors are of interest, as they play a crucial role in cochlear development^10^ and are essential for maintaining neuronal health in the adult cochlea.^11^^,^^12^ Brain-derived neurotrophic factor (BDNF), in particular, has demonstrated neuroprotective and regenerative properties in the auditory system,^13^ offering potential for preserving, protecting, and potentially regenerating SGNs.

Adeno-associated virus (AAV) vectors have gained significant attention as *in vivo* gene delivery tools. This is underscored by the increasing number of market-approved AAV gene therapies (https://www.fda.gov/vaccines-blood-biologics/cellular-gene-therapy-products/approved-cellular-and-gene-therapy-products, https://www.ema.europa.eu/en/medicines/therapeutic-areas-latest-updates) and ongoing clinical trials. Moreover, in the context of inner ear gene therapy, AAV vectors have already shown remarkable success. The world’s first human clinical trial using an AAV vector for this purpose, led by Shu and colleagues, demonstrated the restoration of hearing in children with otoferlin mutations.^14^ Currently, several AAV vector-based phase 1/2 clinical trials (NCT05788536, NCT05821959, NCT05901480) are ongoing, also focusing on treating hereditary hearing loss caused by mutations of the *OTOF* gene through conventional gene addition strategies. Moreover, AAV vectors are explored as auxiliary therapy to improve CI outcomes.^15^

Despite these promising advancements, current AAV vectors are unable to fully exploit the potential of cochlear gene therapy, as several disease-relevant inner ear cell types are transduced with limited efficiency. To date, transduction of HCs in the inner ear of mice at various ages and of non-human primates (NHPs) has been reported, albeit with varying efficiencies.^16^^,^^17^^,^^18^^,^^19^^,^^20^^,^^21^^,^^22^ Similarly, transduction efficiencies for SGNs have been generally low in both murine models and NHPs for natural AAV serotypes and engineered capsids.^19^^,^^23^ Reports of efficient supporting cell (SC) transduction, particularly in adult mice, are promising but also limited.^24^^,^^25^^,^^26^^,^^27^^,^^28^ This is due to the complex and delicate structure of the inner ear, which is notoriously difficult to access, and to capsid-intrinsic features that lower the vector’s ability to transduce distinct cell types.^29^

To address this challenge, AAV capsid engineering is being explored.^30^ One promising approach to redirect vector tropism and improve vector efficacy is the insertion of peptides into the AAV capsid to serve, for example, as receptor-binding ligands. For this purpose, peptides—identified, for example, by phage display library screens—are introduced as oligonucleotides into the *cap* gene. This AAV-specific gene encodes the three capsid proteins, designated viral protein (VP)1, VP2, and VP3, which contribute 5 VP1, 5 VP2, and 50 VP3 subunits to the mature icosahedral AAV capsid.^31^ Preferred insertion sites for peptides are the tips of the highest or second-highest capsid protrusions, located at the 3-fold axis of symmetry, and thus present 60 times per capsid. Depending on the peptide sequence and site of insertion, AAV’s natural receptor-binding features are altered. For vectors with wild-type capsids, interactions with extracellular matrix components, such as heparan sulfate proteoglycan (HSPG) for AAV2, initiate cell transduction and facilitate binding to coreceptors,^32^ promoting the internalization process, for example, through clathrin-dependent endocytosis.^33^ Following entry, viral vector particles are intracellularly processed in a multistep manner, eventually leading to the release of the vector genome from the viral capsid, a process termed uncoating.^34^^,^^35^ Released vector genomes form episomes, which are maintained long-term in non-proliferating cells, including the various cell types of the inner ear, serving as templates for sustained transgene expression.

In the present study, we modified the capsid of AAV2 by inserting a heptamer targeting peptide, flanked by linker amino acids, into each of the 60 capsid protein subunits, thereby altering its cell-surface binding properties and enhancing vector particle uncoating. This change in capsid-intrinsic features enabled AAV.MPI to transduce SGNs in all turns of the cochlea of adult mice. Moreover, transduction of SGNs occurred even at a low dose (1E7 vector particles per inner ear). These novel features establish AAV.MPI as a vector with superior targeting and transduction capabilities. Leveraging these properties, we demonstrated that AAV.MPI-mediated BDNF overexpression effectively protects SGNs following HC loss, underscoring its potential for neuroprotection. Given these findings, we propose that engineered AAV vectors such as AAV.MPI could play a critical role in cochlear gene therapy, offering long-term neuronal preservation that may improve outcomes in CI recipients.

## Results

### Peptide insertion into the AAV2 capsid does not compromise vector production

**Figure 1:**
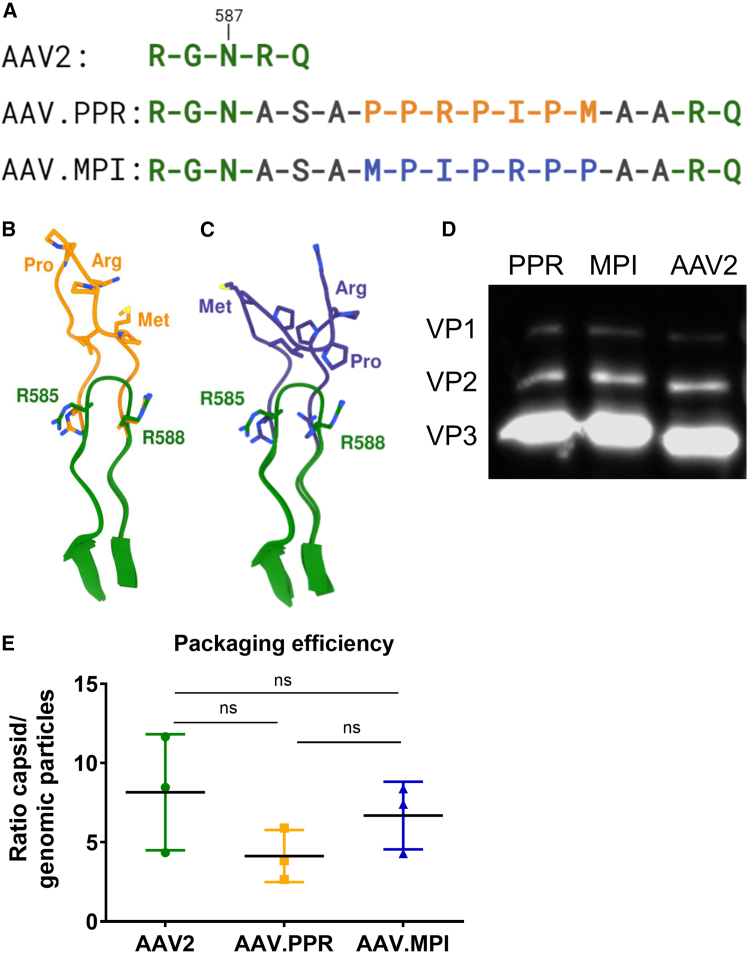
Modeling of PPRPIPM and MPIPRPP peptides as part of VR-VIII (GH12/GH13) of AAV2 VPs and characterization of vector production (A) The PPRPIPM or MPIPRPP peptide was inserted into VP1, VP2, and VP3 of AAV2 at I-587, flanked by alanine-serine-alanine residues at the 5′ end and two alanine residues at the 3′ end. (B and C) Overlay of the tertiary structure of the variable region (VR) VIII loop of (B) AAV.PPR (orange, AlphaFold prediction) or (C) AAV.MPI (blue, AlphaFold prediction) with AAV2 (green, PDB ID 6IH9). (D) Western blot using the B1 antibody, which detects the C-terminus of capsid proteins (VP1: 87 kDa; VP2: 72 kDa; VP3: 62 kDa) in their linear form, to visualize the capsid composition following SDS-PAGE and western blotting of vector preparations (5E9 capsids per lane). (E) Packaging efficiency, expressed as the ratio of capsid titer to genomic particle titer, was determined by ELISA and qPCR, respectively. Scatter dot blot: mean (SD) *n* = 3; ns: not significant; one-way ANOVA with Tukey’s multiple comparison test.

With the goal of identifying peptides with affinity to SCs, we performed a phage display peptide screening against saccule and utricle cultures isolated from neomycin-treated C57BL/6 mice. Two peptides, MPIPRPP and PPRPIPM, with identical amino acid sequences in reverse order, accumulated after four rounds of panning. We inserted the peptides into the tip of the second-highest protrusion of the AAV2 capsid, i.e., position I-587 of the GH12/13 loop (Figure 1A). To improve capsid surface presentation, the peptides were flanked by an alanine-serine-alanine stretch at the 5′ end and two alanine residues at the 3′end (Figure 1A). The capsid variants were named according to the peptide insert as AAV.MPI and AAV.PPR, and were characterized along with the parental AAV2 serotype, which served as a reference.

A structural model prediction analysis suggested that both peptide insertions affect the tertiary structure of the loop (Figures 1B and 1C). Specifically, the proline residues were proposed to cause a slight extension of the peptides from the capsid surface. Moreover, while PPRPIPM in AAV.PPR was predicted to be highly exposed, the MPIPRPP tertiary structure in AAV.MPI faces toward the capsid surface and exposes only its methionine and arginine residues.

We then produced AAV.MPI and AAV.PPR as vectors delivering a self-complementary (sc) Cytomegalovirus (CMV) promoter-driven enhanced Green Fluorescent Protein (eGFP) or dTomato expression cassette and determined capsid composition via SDS-PAGE and western blot analysis. While the ratio of VP1:VP2:VP3 remained unchanged, the modified capsid proteins showed reduced mobility in SDS-PAGE compared to AAV2, as previously observed for other capsid mutants^36^^,^^37^ (Figure 1D). Moreover, the packaging efficiency, defined as the ratio of capsid titer to genomic particle titer, was comparable to that of parental AAV2 (Figure 1E). Thus, we conclude that, for both AAV.PPR and AAV.MPI, peptide insertion neither affects capsid assembly nor vector packaging.

### Capsid variant AAV.MPI, but not AAV.PPR, transduced inner ear cell lines more efficiently than parental AAV2

**Figure 2:**
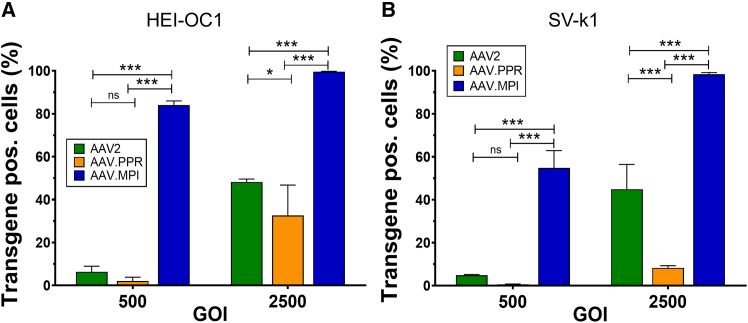
AAV.MPI transduces auditory cell lines more efficiently than AAV.PPR and AAV2 (A and B) Percentage of transgene-expressing HEI-OC1 cells (A) and SV-k1 cells (B) after transduction with the indicated vector preparations at a GOI of 500 and 2,500 at 24 hours post transduction (hpt). Bars, mean (SD), *n* = 3; ns: not significant, ∗*p* < 0.05, ∗∗∗*p* < 0.001; two-way ANOVA with Bonferroni post hoc test. GOI, genomic particles of infection.

Capsid variants were then compared with AAV2 regarding the efficiency of transducing the two most commonly used inner ear cell lines. Specifically, HEI-OC1, a murine cell line of sensory and SC common progenitors originally isolated from the organ of Corti, and SV-k1, derived from murine stria vascularis (SV), were used for these assessments.^38^^,^^39^^,^^40^ AAV.MPI clearly outperformed AAV2 in transducing HEI-OC1 cells (genomic particles of infection [GOI] 2,500; 99% and 48%, respectively; Figure 2A) and SV-k1 cells (GOI 2,500, 98% and 50%, respectively; Figure 2B). In contrast, transduction efficiencies of AAV.PPR were lower in HEI-OC1 cells (Figure 2A; GOI 2,500; 33%) and SV-k1 cells (Figure 2B; GOI 2,500; 8%) compared with the parental AAV2 vector.

### AAV.MPI and AAV.PPR peptide insertion into the AAV2 capsid reduce HSPG binding

**Figure 3:**
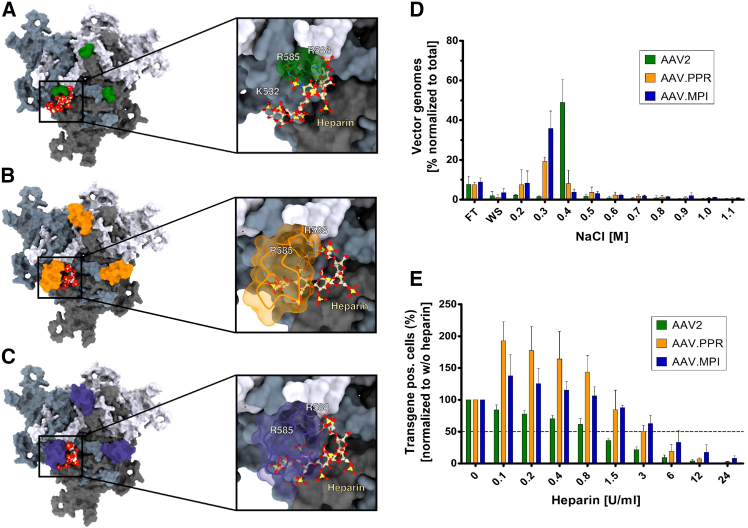
AAV.MPI and AAV.PPR exhibit reduced affinity to HSPG compared with AAV2 (A–C) Surface model of heparin docking in the heparan sulfate proteoglycan-binding pocket of AAV2 (A) and the corresponding region of AAV.PPR (B) and AAV.MPI (C) VP trimers, using VP3 as the basis. Additionally, close-ups of heparin interactions with the VR-VIII loop, including crucial residues, are shown. (A) Heparin contacts R585, R588, R484, and K532, with the HSPG-binding motif constituted by two AAV capsid subunits. Panel A was adapted from Meumann et al.^73^ and is shown for comparison. (B and C) For AAV.MPI and AAV.PPR, the model predicts heparin binding to R585 and R588 but interference with heparin binding to K532 and R484 due to the peptide insertion between N587 and R588, i.e., at I-587. (D) Heparin affinity chromatography. AAV.PPR, AAV.MPI, and AAV2 vectors were loaded onto a heparin affinity column, and flow-through (FT) and wash (WS) fractions were collected. AAV vectors were gradually eluted with increasing concentrations of sodium chloride (NaCl) (0.2–1.1 M) in PBS/MgCl_2_/KCl. Vector genome-containing particles in the collected fractions were quantified by qPCR. Bars, mean (SD), *n* = 3. (E) Heparin competition assay. AAV-PPR, AAV.MPI, and AAV2 vectors were pre-incubated with increasing heparin concentrations (0–24 U/ml) for 30 min at RT and then administered to HEI-OC1 cultures at a GOI of 500. At 48 hpt, eGFP expression levels were measured by flow cytometry and normalized to no-heparin controls. Bars, mean (SD), *n* = 3. GOI, genomic particles of infection.

Peptide insertions at position I-587 within AAV2 capsid proteins separate the two arginine (R) residues, R585 and R588, which are part of the HSPG receptor-binding motif of AAV2. Additional residues of this motif—provided by an adjacent subunit—include R484, R487, and lysine (K)532 (Figure 3A).^41^^,^^42^ Depending on the sequence of the inserts, the capsids may become blind to HSPG, allowing the newly inserted peptide to define the tropism of the engineered particle.^43^ In general, the extent of interference with the natural infection process depends on both the specific peptide sequence and the three-dimensional structure formed at the insertion site. Based on this, the interaction of AAV.MPI and AAV.PPR with heparin, a soluble analog of HSPG, was modeled. The results suggest that, in contrast to AAV2, only residual binding is feasible, predominantly mediated by R585 and R588 (Figures 3B and 3C).

To confirm the predicted change in binding affinity, we performed a heparin affinity chromatography assay (Figure 3D). Indeed, AAV2 differed strongly from AAV.MPI and AAV.PPR in the number of particles retained on the heparin column. When eluting bound particles using an increasing salt gradient, AAV2 vector particles were predominantly eluted at 0.4 M sodium chloride (NaCl), whereas AAV.MPI particles were eluted at 0.2–0.3 M NaCl (15% and 27% of total eluted particles, respectively), and AAV.PPR particles were mostly eluted at 0.3 M NaCl (19% of total eluted particles).

To determine the functional consequences of reduced heparin-binding affinities on cell transduction, we conducted a heparin competition assay on HEI-OC1 cells (Figure 3E). Interestingly, for both AAV.MPI and AAV.PPR, the addition of a low concentration of heparin increased transduction efficiencies, whereas this low concentration already negatively affected AAV2 vector-mediated transductions. The presence of approximately 1 U/ml of heparin was sufficient to reduce the transduction efficiency of AAV2 vector particles by half (IC_50_). In contrast, both capsid variants demonstrated an IC_50_ of ∼3 U/ml of heparin, indicating a 3-fold higher tolerance (Figure 3E). Thus, consistent with the structural models, the experimental findings corroborate that, compared to AAV2, the insertion of either PPRPIPM or MPIPRPP reduces the affinity of capsids for HSPG, thereby altering the cell surface-binding features. This suggests that cell transduction by AAV.MPI and AAV.PPR capsids is significantly less dependent on HSPG compared with AAV2 (if at all) and is instead mediated by the inserted peptide.

### AAV.MPI capsid demonstrates enhanced transduction efficiency in the mature cochlea *in vivo*

**Figure 4:**
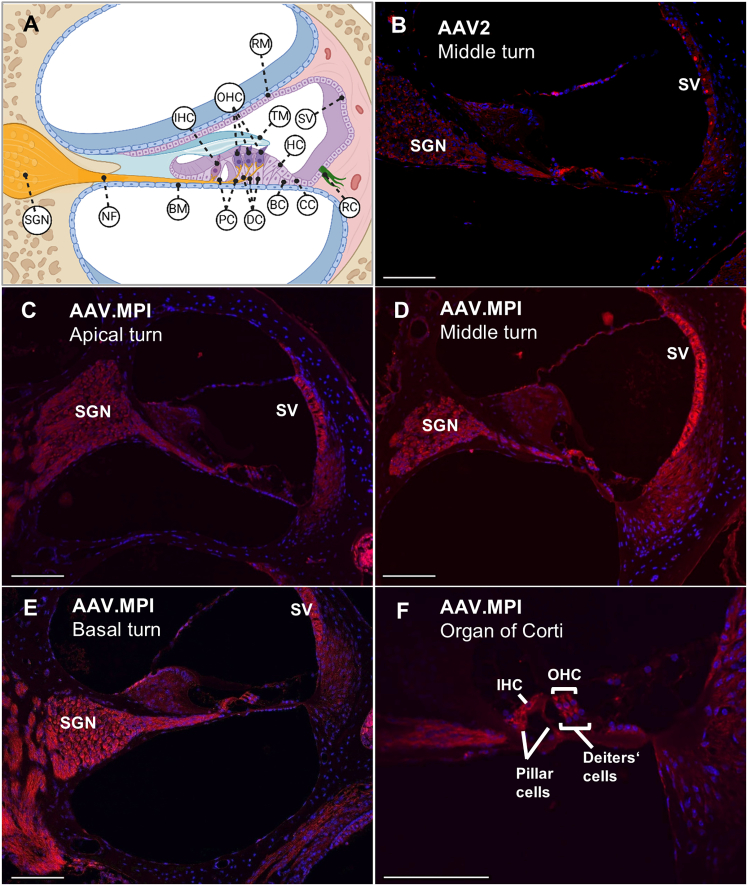
AAV.MPI capsid efficiently transduces multiple cell types in the adult mouse cochlea One-month-old C57BL/6J mice were injected with AAV2-CMV-dTomato or AAV.MPI-CMV-dTomato vectors via canalostomy (PSCC) or left untreated (Figure S1), and cochleae were analyzed 7 days post injection (dpi). (A) Schematic of the anatomy of a cochlear cross-section. IHC, inner hair cell; OHC, outer hair cells; DC, Deiters’ cells; PC, pillar cells; HC, Hensen’s cells; BC, Boettcher cells; CC, Claudius’ cells; BM, basilar membrane; TM, tectorial membrane; RM, Reissner membrane; NF, nerve fibers; SGN, spiral ganglion neurons; SV, stria vascularis; RC, root cells. Created with Biorender. (B) Representative cross-section from a cochlea injected with AAV2 vectors (8.6E8 vg) (C–F) Representative cross-section from a cochlea injected with AAV.MPI vectors (1.0E9 vg) showing the apical (C), middle (D), and basal (E) turns of the cochlea, and a close-up of the organ of Corti (F). Red indicates dTomato-expressing cells, and blue indicates DAPI-stained nuclei. Scale bars, 100 μm. vg, vector genome-containing AAV particles.

**Figure 5:**
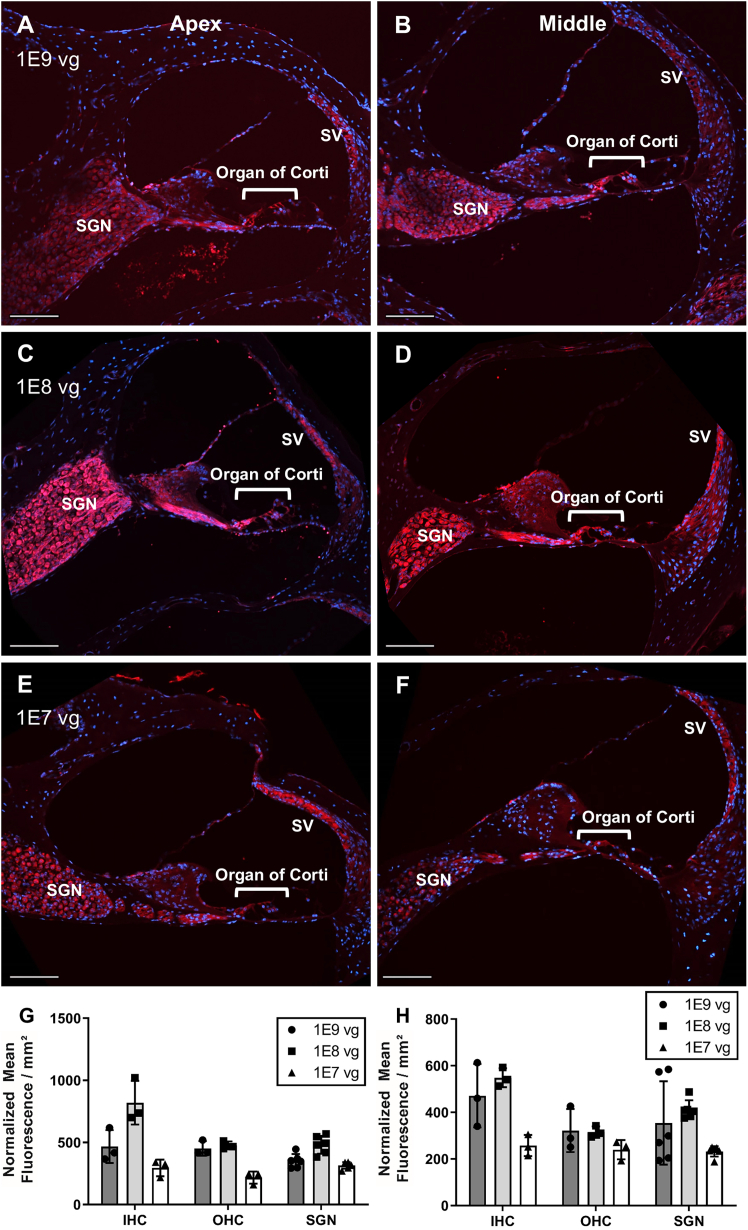
AAV.MPI shows SGN transduction in *in vivo*-injected cochleae at different doses One-month-old C57BL/6J mice were injected with AAV.MPI-CMV-dTomato vector at doses of 1E9 vg (A–B), 1E8 vg (C–D), or 1E7 vg (E–F) using canalostomy (PSCC), and cochleae were analyzed 7 days post injection. (A–F) Representative cross-sections of the apical (A, C, E) and middle (B, D, F) turns of the cochleae. (G ad H) Quantification of the normalized mean fluorescence of the vector-encoded reporter protein per mm^2^ from three cross-sections for each condition in IHCs, OHCs, and SGNs of the apical (G) and middle (H) cochlear turns. Bars, mean (SD). Red indicates dTomato expressing cells, and blue indicates DAPI-stained nuclei. Scale bars, 100 μm. vg, vector genome-containing AAV particles.

Since AAV.PPR showed significantly lower transduction efficiency in our model cell lines (Figures 2A and 2B) and no meaningful difference in residual binding to HSPG compared with AAV.MPI (Figure 3), we focused on AAV.MPI for further analysis. We assessed overall tropism by local delivery via canalostomy through the posterior semicircular canal (PSCC) to 1-month-old wild-type C57BL/6 mice, using AAV2 as a reference. One week post-injection, cochleae were isolated, sectioned, and imaged (Figures 4B–4F). Cochlea of non-treated animals were processed in parallel and served as negative controls (Figure S1). While fluorescent reporter gene expression in AAV2-injected inner ears was high in inner HCs (IHCs) but low in outer HCs (OHCs) and SGNs (Figure 4B), AAV.MPI mediated robust transduction of IHCs, OHCs, and SGNs (Figure 4D). In addition, AAV.MPI transduced pillar and Deiters’ cells, root cells, and the SV across all cochlear turns (Figures 4C–4F). To define a dose range required for efficient SGN transduction, we next compared AAV.MPI-mediated dTomato expression across decreasing vector doses. Notable SGN transduction could be detected at doses as low as 1E7 vector particles, as shown here for the apical and middle turns of the mature murine cochlea (Figures 5A–5F). Quantification of mean fluorescence intensities in IHCs, OHCs, and SGNs showed enhanced transduction efficiency at 1E8 vector particles, consistent with qualitative histological observations (Figures 5G and 5H). To further quantify transgene expression independently of histological analysis, we performed a luciferase assay on cochlear lysates from mice injected with AAV.MPI-CMV-Renilla luciferase at vector doses of 1×10^9^, 1×10^7^, and 1×10^5^ vg per mouse (Figure S2). Luciferase activity, measured at 5 days post-injection (dpi), showed a dose-dependent, non-linear increase, confirming robust AAV.MPI-mediated expression in the adult murine cochlea, even at remarkably low doses (Figure S2).

A few recent studies have reported the detection of AAV vectors in the contralateral ear or even systemically following local inner ear injection.^44^^,^^45^^,^^46^ As part of the safety profiling of AAV.MPI, we subsequently determined the biodistribution of AAV2 and AAV.MPI at the vector genome level in the injected cochlea, contralateral cochlea, brain, and liver at 7 days post-PSCC injection. All samples from the contralateral ear—except one from an AAV2 vector-injected inner ear—as well as brain and liver samples were negative compared with non-injected mouse controls (Figure S3). Moreover, AAV.MPI administration via PSCC did not affect hearing or balance in mice (Figure S4).

In summary, the insertion of MPIPRPP at I-587 confers the AAV2 capsid with tropism for SGNs, which are typically difficult to transduce. Moreover, the peptide insertion enables the AAV2 capsid variant to transduce the SV, HCs, and pillar and Deiters’ cells. Notably, AAV.MPI transduces adult mice—a challenging target population—and achieves remarkable efficacies even at low vector doses.

### AAV.MPI uncoats earlier and more efficiently than AAV2 *in vitro* and *in vivo*

**Figure 6:**
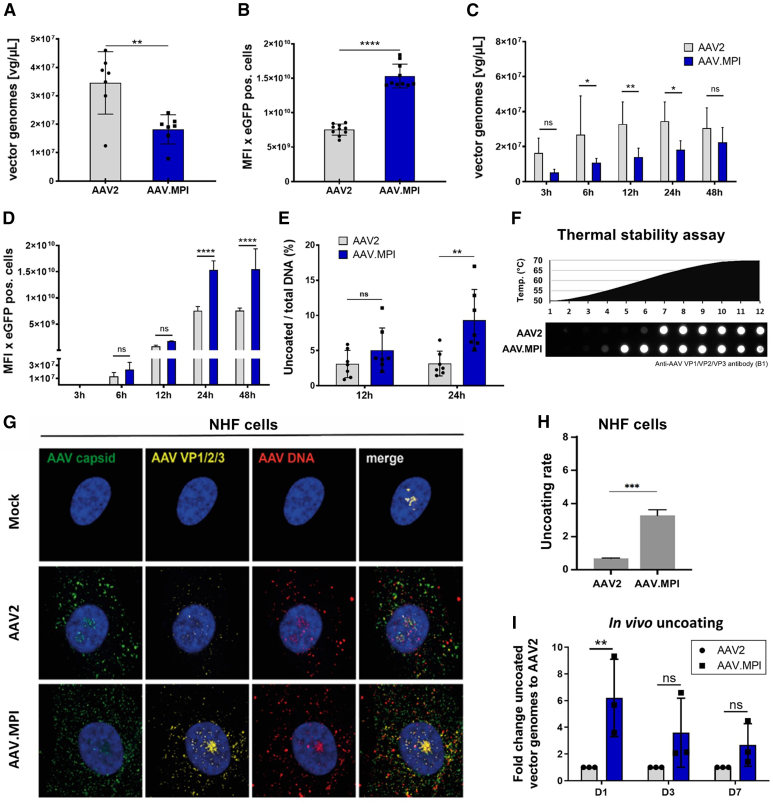
Compared to AAV2, AAV.MPI shows distinct features, including enhanced uncoating, that lead to improved transduction efficiency (A) Intracellular vector copy numbers of AAV2 and AAV.MPI in HEI-OC1 cells after 24 h (GOI 5,000). DNA was extracted from whole lysates and analyzed by qPCR using transgene-specific primers. Bars, mean (SD), *n* = 3, ∗∗*p* < 0.01, unpaired *t* test. (B) Transgene expression of AAV2 and AAV.MPI on HEI-OC1 cells after 24 h (GOI 5,000), analyzed by flow cytometry. Mean fluorescence intensity (MFI, median) was multiplied by the number of transgene-positive cells. Bars, mean (SD), *n* = 3, technical triplicates, ∗∗∗∗*p* < 0.0001, unpaired *t* test. (C) Amount of intracellular vector copies of AAV2 and AAV.MPI in HEI-OC1 cells at the indicated time points (GOI 5,000). Genomic DNA was extracted from whole lysates and analyzed by qPCR using transgene-specific primers. Bars, mean (SD), *n* = 3, ns: not significant, ∗*p* < 0.05, ∗∗*p* < 0.01, two-way ANOVA with Bonferroni post hoc test. (D) Transgene expression of AAV2 and AAV.MPI in HEI-OC1 cells at the indicated time points (GOI 5,000), analyzed by flow cytometry. MFI median was multiplied by the number of transgene-positive cells for each time point and vector. Bars, mean (SD), *n* = 3, ns: not significant, ∗∗∗∗*p* < 0.0001, two-way ANOVA with Bonferroni post hoc test. (E) *In vitro* uncoating in the nuclear fraction of HEI-OC1 cells treated with AAV2 and AAV.MPI after 12 and 24 h. Isolated DNA was treated with T5 exonuclease, and the ratio of episomal to total DNA was analyzed by qPCR using transgene-specific primers. Bars, mean (SD), *n* = 3, technical triplicates, ns: not significant, ∗∗*p* < 0.01, two-way ANOVA with Bonferroni post hoc test. (F) Capsid destabilization assay for AAV2 and AAV.MPI. Vector preparations were incubated for 30 min at the specified temperatures, followed by native dot blot using the B1 antibody to detect disintegrated capsids. Representative image of *n* = 3. (G) Co-detection of vector DNA with vector capsids and vector capsid proteins. NHF cells were transduced with either AAV2 or AAV.MPI (GOI 20,000). At 24 hpt, cells were fixed and processed for multicolor IF analysis combined with FISH. Intact capsids (green) or capsid proteins (yellow) were detected using either an antibody against intact AAV2 capsids or an antibody against VP1, VP2, and VP3. AAV2 DNA (red) was detected with an Alexa Fluor (AF) 647-labeled, amine-modified DNA probe that binds to the AAV2 genome. Nuclei were counterstained with DAPI (blue). (H) Image-based quantification of the complete uncoating rate of AAV2 and AAV.MPI, determined as the ratio of capsid-DNA+/capsid+DNA+ of 50 individual cells for each vector. Bars, mean (SD), ∗∗∗*p* < 0.001, unpaired *t* test. (J) Indirect uncoating assay of vector-treated (8E8 vg) murine cochlea at 1, 3, and 7 days post injection. Cochleae were isolated, DNA was extracted and treated with T5 exonuclease or mock-treated. Samples were analyzed by qPCR using transgene-specific primers and normalized to horseradish peroxidase (*hprt*) and control samples from not injected mice. Bars, mean (SD), fold-change of uncoating in AAV.MPI injected cochleae to AAV2, *n* = 3, ns: not significant, ∗∗*p* < 0.01, two-way ANOVA with Bonferroni post hoc test. GOI, genomic particles of infection.

To decipher possible reasons for the improved performance of AAV.MPI compared with AAV2, we aimed to determine differences in uptake efficiency. For this, intracellular vector particles were quantified 24 h post-transduction (hpt) of HEI-OC1 cells by qPCR. Interestingly, despite administration of equal GOI, intracellular vector copy numbers for AAV.MPI were found to be half compared to those of AAV2 (1.8E7 and 3.5E7 vg/μl, respectively; Figure 6A). Despite this lower intracellular vector copy number following cell line transduction, significantly higher transgene expression levels were achieved with AAV.MPI (Figure 6B).

To explore the transgene expression kinetics of AAV.MPI, we correlated intracellular vector copy numbers with transgene expression levels in HEI-OC1 cells at different time points—3, 6, 12, 24, and 48 hpt (Figure 6C). At 6, 12, and 24 hpt, AAV.MPI showed significantly lower intracellular vector genomes than AAV2 (Figure 6C). Transgene expression was already detectable in both AAV.MPI and AAV2-transduced cultures at 6 hpt and increased until 24 hpt (Figure 6D). However, AAV.MPI-transduced cultures showed higher transgene expression levels at all time points despite lower VCNs, indicating more efficient intracellular processing of AAV.MPI compared with AAV2 vector particles.

Detection of vector genomes within the cell does not discriminate between uncoated vector genomes, which can serve as templates for transcription, and encapsulated vector genomes. We therefore conducted an indirect uncoating assay using T5 exonuclease, which depletes linear double-stranded DNA, leaving only inherently resistant episomes. These episomes represent the final configuration of the vector genome.^35^ Nuclear fractions from HEI-OC1 cells transduced with either AAV2 or AAV.MPI particles were extracted at 12 and 24 hpt and either exposed or not exposed to exonuclease treatment, followed by quantification of vector genome sequences using qPCR. The percentage of episomal DNA, given as the ratio of vector genomes in T5 exonuclease-treated vs. non-treated subfractions, was 1.5- and 3-fold higher for AAV.MPI compared with AAV2 at 12 and 24 hpt, respectively, although statistical significance was reached only at 24 hpt (Figure 6E). These results correlate with the respective relative transgene expression (% positive cells × MFI) values (Figure 6D), suggesting that AAV.MPI uncoats more efficiently in HEI-OC1 cells.

Although the mechanisms underlying the uncoating process are still largely unknown, we previously reported that reduced capsid stability correlates with increased uncoating efficiency.^35^^,^^47^ Therefore, we determined capsid stability for AAV2 and AAV.MPI by exposing vector particles to a temperature gradient. Disassembled vector capsids were subsequently detected by immunoblotting using the B1 (anti-AAV VP1/2/3) antibody. Indeed, AAV.MPI capsids were found to disassemble at a lower temperature compared with AAV2 capsids (57.9°C and 63.4°C, respectively; Figure 6F), consistent with our previous findings.

Since uncoating of viral vectors is generally considered a rate-limiting intracellular step in the transduction process, we conducted a more detailed investigation of the uncoating profile of AAV.MPI compared with AAV2. Uncoating occurs in a stepwise process, beginning in the cytosol and completing in the nucleolus, supported by structural reorganization of the nucleolus.^48^ These recently published observations for AAV2 were based on the combination of immunofluorescence analysis to detect intact AAV2 capsids (capsid+) and capsid proteins (VP1/2/3+), along with fluorescence *in situ* hybridization to detect AAV2 genomes (DNA+).^48^ Using this established assay, we compared the uncoating of AAV.MPI and AAV2 vector particles in normal human fibroblast (NHF), which served as the model system in the aforementioned study, at the most relevant time point, 24 hpt (Figure 6G). Consistent with our previous results,^48^ complete uncoating of AAV2 was dependent on nucleolar reorganization, indicated by AAV2 DNA+ nucleoli that were negative for AAV2 intact capsids (capsid−) or positive for VP1/2/3, predominantly present in more dispersed nucleoli. However, unlike AAV2, we observed that all AAV.MPI capsid−/DNA+ nucleoli were also AAV.MPI VP1/2/3+ and DNA+, regardless of nucleolar structure. As no difference was detected in the ratios of dense to dispersed nucleoli in AAV2- and AAV.MPI-infected cells (Figure S5), complete uncoating of AAV.MPI appears to occur independently of nucleolar structure—and hence possibly independently of cell cycle progression—differing substantially from AAV2. Moreover, image-based quantification showed that the rate of complete uncoating (capsid-DNA+/capsid+DNA+) in nucleoli after 24 h was approx. 3-fold higher for AAV.MPI than for AAV2 (Figure 6H). Based on these findings, we hypothesize that complete uncoating of AAV.MPI occurs more efficiently than that of AAV2 in cell lines.

Since most cells of the inner ear are post-mitotic, the use of immortalized otic cell lines to analyze intracellular barriers may only partly resemble the processes occurring *in vivo*. Therefore, we assessed the uncoating efficiency of AAV2 and AAV.MPI directly *in vivo* in the cochleae of healthy C57BL/6J mice at 1, 3, and 7 days post-injection into the PSCC. Consistent with our *ex vivo* results in HEI-OC1 and NHF cells, we detected considerably more episomal vector genomes for AAV.MPI compared with AAV2 at all tested time points (Figures 6J and S6), indicating that AAV.MPI also uncoats more efficiently *in vivo* than AAV2.

AAVR is an essential receptor for most AAV serotypes to infect human cells and mouse tissues.^49^^,^^50^ Recent research identified AAV2’s binding site for AAVR at the second-highest protrusion, proximate to the 3-fold symmetry axis.^51^^,^^52^ Since the capsid insertion in AAV.MPI is located adjacent to the AAVR binding site, we investigated whether AAV.MPI differs from AAV2 in its reliance on AAVR for cell entry. Using HEK293-AAVR-KO and HeLa-AAVR-KO cells, alongside the parental AAVR wild-type cells as models, we compared AAV.MPI and AAV2 at increasing vector particle-per-cell ratios (Figure S7). In the absence of AAVR, AAV2’s transduction efficiency dropped to background levels, as expected. Similarly, AAV.MPI transduced both parental cell lines but failed to transduce AAVR knockout cells, highlighting the essential role of AAVR for both AAV2 and AAV.MPI, at least in these otherwise highly permissive cell lines.

### BDNF gene transfer with AAV.MPI protects SGN degeneration in deafened mice

**Figure 7:**
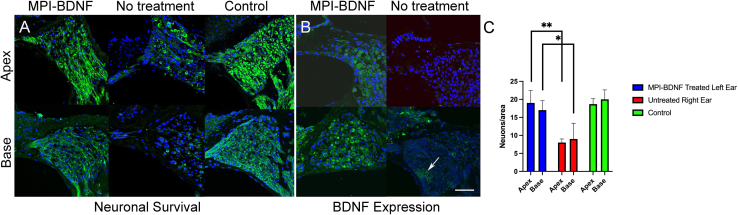
Delivery of BDNF by AAV.MPI results in enhanced spiral ganglion survival Adult C57BL/6J mice were deafened using an aminoglycoside and loop diuretic and subsequently received AAV.MPI-BDNF (9E8 vg per inner ear) via canalostomy (PSCC) three months post-deafening. Cochleae were collected three months after vector administration. (A) TUJ-1 positive neurons (green) with associated neurites are seen in representative cochlear cross-sections from both the apical and basal turn of deafened AAV.MPI-BDNF-treated mice (left). Few surviving neurons are observed in the deafened, vector-untreated contralateral spiral ganglion (middle). Degeneration is more marked in the untreated basal turn, with degeneration of all neurites but only a few surviving neuronal cell bodies. In contrast, the untreated wild-type control cochlea (right) shows abundant neurons and intact neurites throughout. (B) Representative immunofluorescent staining with an anti-BDNF antibody shows robust labeling on the AAV.MPI-BDNF-treated side, with minimal fluorescent signal on the vector-untreated side (arrow). (C) There is a statistically significant survival of neurons in both the apical (*p* < 0.01) and basal (*p* < 0.05) spiral ganglion of deafened AAV.MPI-BDNF-treated mice compared with deafened, vector-untreated mice. Bars, mean (SEM), *n* = 5 mice per cohort, ∗*p* < 0.05, ∗∗*p* < 0.01 two-tailed unpaired *t* test with Welch’s correction.

Exogenous expression of BDNF after HC loss has been reported to promote neural survival, potentially providing a therapeutic option to maintain SGN function for people using CI.^10^^,^^13^^,^^15^^,^^53^^,^^54^ However, AAVs have not been used in this context to ectopically express BDNF directly in SGNs of adult mice. To evaluate this possibility, targeting of SGNs by AAV.MPI in the absence of HCs was confirmed using a dTomato-encoding AAV.MPI in mice with induced HC loss (Figure S8A). HC loss was confirmed by auditory brainstem response (ABR) measurements (Figure 6B). We then produced AAV.MPI as a vector encoding BDNF controlled by a CMV promoter (Figure S9). For the proof-of-concept, mice were deafened with a combination of an aminoglycoside and a loop diuretic, resulting in loss of all residual hearing (Figure S8B). Part of this cohort received AAV.MPI-BDNF (9E8 vg per inner ear) 3 months post-deafening. Three months after AAV.MPI-BDNF treatment, mice were euthanized, followed by collection of cochleae (Figure 7A). To assess SGN tissue morphology, histopathological analysis of cochleae from “deafened AAV.MPI-BDNF treated,” “deafened, vector-untreated,” and “untreated wildtype control” mice was performed by immunostaining residual SGNs with the TuJ1 antibody. Normal SGN morphology was preserved in healthy control mice, as expected (Figure 7A). However, substantial SGN degeneration was observed in the “deafened, vector-untreated” cohort 6 months post-deafening, with only a few surviving neurons in the untreated contralateral spiral ganglion. Degeneration was more marked in the untreated basal turn, with loss of all neurites except for a few surviving neuronal cell bodies (Figure 7A). Quantification of SGNs confirmed the significant protective effects in both basal and apical cochlear turns of mice receiving AAV.MPI-BDNF (blue), restoring SGN numbers to levels comparable with healthy controls (green), as compared to deafened, untreated mice (red) (Figure 7C). To confirm BDNF expression in the cochleae, we performed histological analysis of “deafened, vector-untreated” and “deafened, AAV.MPI-BDNF-treated” cochleae (Figure 7B). As expected, minimal BDNF immunofluorescent signal was detected in the untreated control ear, whereas AAV.MPI-BDNF treatment resulted in BDNF expression in SGNs (Figures 7C and S8). This confirms the successful delivery of BDNF-encoding vector genomes by AAV.MPI and the subsequent expression of BDNF in the targeted cochlear regions following PSCC administration of AAV.MPI.

## Discussion

Gene therapy for patients with hearing loss caused by mutations in the *OTOF* gene marks a pioneering step in targeting cochlear HCs for hearing restoration.^14^^,^^55^ Similar to other organs, transduction efficacy of HCs has been improved by switching from AAV vectors with naturally occurring serotype capsids to synthetic or engineered capsids. The first example of this approach was AAV-Anc80L65, a synthetic capsid developed by *in silico* reconstruction.^56^ However, SNHL can result from dysfunction or degeneration not only of HCs but also of various other inner ear cells, such as SCs, cells of the SV, and SGNs, among others.^57^ Here, we report the development of a new capsid variant, AAV.MPI, which represents the first AAV vector capable of efficiently transducing SGNs in adult mice. Moreover, AAV.MPI additionally transduces other therapeutically relevant inner ear cell types—SV, HCs, and pillar and Deiters’ cells—thereby considerably expanding the repertoire of AAV vectors for inner ear gene therapy.

The peptide MPIPRPP, which we inserted as a receptor-binding ligand into I-587 of all 60 capsid subunits, was derived from phage display biopanning. This method allows screening of a significantly larger set of peptides compared with AAV peptide display approaches.^58^^,^^59^^,^^60^ In the context of the AAV2 capsid, the peptide MPIPRPP mediated efficient transduction of SGNs, SCs, and SVs in all turns of the cochlea (Figure 4) in the mature inner ear. This feature is crucial in light of potential later application in humans, due to developmental differences in hearing onset between mice and humans. While hearing matures in mice at approximately postnatal day 12,^61^^,^^62^ the human inner ear is already fully developed by the 26^th^ week of gestation.^63^ However, mature inner ear cells, particular OHCs and SGNs, were so far been transduced only inefficiently by AAV vectors^28^^,^^57^ and other delivery systems.^64^^,^^65^^,^^66^^,^^67^^,^^68^ For example, while the above-mentioned Anc80L65 transduces up to 90% of OHCs in neonatal mice via the round window membrane, it reaches only 60%–85% of OHCs at 5.66–11.32 kHz and 20%–30% at other frequencies in adult mice via canalostomy (PSCC).^26^

An additional key advantage of AAV.MPI is its ability to transduce the mature cochlea even at low vector doses (Figures 5 and S2). To validate the histology-based quantification of transgene expression (Figures 5G and 5H), we replaced dTomato with Renilla luciferase as the transgene and performed a quantitative enzymatic assay on cochlear lysates with an expanded dose range that included a vector dose of 1E5 vg per mouse (Figure S2). The results confirmed the remarkable efficacy of AAV.MPI even at low vector doses, as reported in the detailed histological analyses of cochlear sections. This efficacy, combined with the ability to transduce inner ear cells of mature cochlea, clearly distinguishes this novel capsid from current benchmarks, which are commonly applied at doses of 1E9 vg in neonatal animals.

Moreover, in a deafened mature mouse cochlea, AAV.MPI also transduced SGNs (Figure S8A), thereby excluding the possibility that AAV.MPI depends on retrograde axonal transport through HCs to reach SGNs. Retrograde transport of AAV vectors typically involves viral particles being taken up by axonal terminals at the injection site and then transported back to the neuronal cell soma, where the neuron is subsequently transduced.^69^^,^^70^ By confirming direct AAV.MPI expression in SGNs without intermediary transport through HCs (Figure S8A), we establish a more precise understanding of AAV.MPI’s transduction pathway and efficacy within the inner ear.

In order to better understand which intracellular barriers interfere with efficient AAV vector-mediated cell transduction in the auditory system, we performed a comprehensive analysis of AAV.MPI’s host cell interactions. Cell transduction begins with the attachment of the vector to the cell surface, mediated by receptor binding. In the case of AAV2, this process is facilitated by its interaction with HSPG on the cell surface. Compared to AAV2, AAV.MPI showed a substantial reduction in HSPG binding, which is likely a result of its inability to interact with K532 and R484, as predicted by structural analysis. AAV.PPR, which presents the same peptide sequence in reverse order, showed HSPG binding characteristics comparable to AAV.MPI. However, it differed significantly from AAV.MPI in transduction efficiency in the murine progenitor cell lines HEI-OC1 and SV-k1, arguing that MPIPRPP (used in the case of AAV.MPI) and PPRPIPM (used in the case of AAV-PPR) differ in the internalization receptor to which they bind in the context of the AAV capsid.

Binding to the internalization receptor may be supported by the residual affinity of AAV.MPI to HSPG (Figure 3), as observed by our research group and others for other capsid-engineered AAV vectors.^35^^,^^71^^,^^72^^,^^73^ For instance, Woodard and colleagues reported that residual AAV binding to HSPG at the inner limiting membrane within the eye following intravitreal administration is required to mediate passage through this barrier, resulting in enhanced retinal transduction.^71^ This finding was confirmed in NHPs by Kellish and colleagues.^72^ Similarly, AAV-NN and AAV-GL, two AAV2-based capsid-engineered vectors that maintained residual HSPG binding capability, showed enhanced transduction efficiency of photoreceptors following intravitreal administration.^74^ Further examples include AAV-VSSTSPR, selected to enable efficient transduction of dendritic cells,^35^ and MLIV.K and MLIV.A, developed for improved transduction of hepatocytes following intravenous administration.^73^

When comparing AAV.MPI to AAV2 for *in vitro* transduction of the otic cell line HEI-OC1, a lower vector copy number was detected, which is likely a consequence of the reduced affinity for HSPG.^43^ Despite the lower number of intracellular vector copies, 2-fold higher transduction efficiencies (% positive × MFI) were achieved after 24 h in HEI-OC1 cells (Figures 6A and 6B). Moreover, AAV.MPI clearly outperformed AAV2 *in vivo*. Both findings argue for an altered host-vector interaction also at a post-entry level. Our analyses identified vector uncoating as a key step, as AAV.MPI demonstrated more efficient uncoating both *ex vivo* and *in vivo* compared to AAV2 (Figure 6). Moreover, AAV.MPI differs from AAV2 in its ability to uncoat independently of nucleolar structure. Sutter et al. correlated structural reorganization of nucleoli with cell cycle progression, discovering that AAV2 uncoating was obstructed in dense nucleoli during the G1 phase.^48^ However, complete AAV2 uncoating was observed predominantly in dispersed nucleoli, which are found during the G2/S phase, arguing for a cell cycle-dependent uncoating of AAV2. Based on our new findings, it is tempting to speculate that AAV.MPI uncoats independently of cell cycle progression. While AAV2 can transduce both proliferating and non-dividing cells, transduction efficiency tends to be lower in quiescent cells,^47^^,^^48^ a likely disadvantage in the inner ear. The observed independence of the capsid-engineered vector AAV.MPI from structural reorganization of nucleoli, and thus possibly from cell cycle progression, positions AAV.MPI as a unique candidate for efficient gene delivery to post-mitotic cells in the inner ear, offering potential advantages in therapeutic efficacy.^75^^,^^76^

While neurotrophic factor administration has been shown to protect SGNs and HCs, direct protein delivery is limited by short half-lives and infection risks.^53^^,^^54^^,^^75^^,^^76^^,^^77^ In contrast, AAV vector-mediated neurotrophic factor delivery enables sustained expression from a single treatment, making it a more viable approach for long-term neuroprotection.^78^ AAV2-BDNF delivery has been shown to restore synapses, improve ABR wave I amplitudes, and protect SGNs after noise-induced damage.^79^ Additionally, adenoviral-mediated delivery of BDNF or neurotrophin-3 (NT-3) to the inner ear has been associated with increased SGN survival.^80^

Our study builds upon this body of work but reveals the potency of using an application-tailored AAV capsid variant for this purpose. AAV.MPI, our capsid-engineered variant, delivered the neurotrophic transgene directly to SGNs, the actual target cells (Figure 7). AAV.MPI-mediated BDNF expression resulted in robust SGN preservation across all cochlear regions following HC loss, suggesting that this vector effectively delivers functional transgenes directly (not via retrograde transport) to SGNs (Figure S8A). While AAV vector-based gene therapy approaches for cochlear protection have been explored,^81^^,^^82^ AAV.MPI efficiently transduces SGNs even at low vector doses, a particularly promising feature for clinical translation. The ability to achieve robust SGN protection at reduced vector doses may also mitigate concerns related to vector-associated toxicity and immune responses, which have been reported for high-dose AAV vector administrations.^81^

In summary, we introduce AAV.MPI as a capsid-engineered vector with enhanced SGN targeting and transduction efficiency. Compared to its parental serotype AAV2, AAV.MPI demonstrates improved cell attachment and intracellular processing, contributing to its high efficacy in transducing SGNs even at low doses. These characteristics make AAV.MPI a promising candidate for delivering therapeutic transgenes, including neurotrophic factors such as BDNF, to support long-term SGN survival. Our findings establish a foundation for the use of AAV.MPI in cochlear gene therapy strategies aimed at preserving neuronal health and function, with potential applications in combination with CIs.

## Material and methods

### Animals

All procedures were approved by the University of Kansas Medical Center (KUMC) Institutional Animal Care and Use Committee 2018–2442. One-month-old C57Bl/6 mice were used for all experiments, unless otherwise stated.

### AAV vector production

For the generation of AAV.MPI and AAV.PPR encoding helper plasmids for AAV vector production, sense and antisense oligonucleotides representing the respective peptide sequencing, flanked by linkers and restriction enzymes cutting sites for AscI and MluI, were synthesized (Eurofins Genomics GmbH) and inserted into pRC’99^83^^,^^84^ to produce pRC.MPI and pRC.PPR, respectively. Vector production and purification were performed as previously described.^47^ For AAV2, the helper plasmid pRC^83^ and for AAV.MPI and AAV.PPR the corresponding helper plasmids pRC.MPI and pRC.PPR, respectively, were used in addition to plasmids containing a sc CMV-eGFP, scCMV-dTomato, scCMV-Renilla luciferase, or scCMV-human BDNF expression cassette,^85^ and the adenoviral helper plasmid pXX6.^86^

After purification by discontinuous iodixanol gradient ultracentrifugation, filter column ultracentrifugation (Amicon Ultra Centrifugal Filter Units, Merck Millipore) was performed to further purify, concentrate, and re-buffer vector preparations in phosphate-buffered saline (PBS)/M/K (1× PBS/1mM MgCl_2_/2.5 mM KCl).

### AAV vector characterization

Genomic particle titers were determined using absolute qPCR quantification with a LightCycler 96 real-time PCR system (Roche) using transgene- or promoter-specific primers (eGFP_fw: CACAACGTCTATATCATGGC; eGFP_rev: TGTGATCGCGCTTCTC, dTomato_fw: GCCACTACCTGGTGGAGTTCAAG, dTomato_rev: GTTCCACGATGGTGTAGTCCTCG, CMV_fw: AGTATTTACGGTAAACTGCCC, CMV_rev: GCGATGACTAATACGTAGATGTAC, BDNF_fw: GATAGCATCAGCGAGTGGGT, BDNF_rv: CCCTCTTTGGTGTAGCCCAT) and the corresponding vector plasmid standard curve. Capsid titers were quantified using the AAV2 Titration ELISA Kit (Progen). For western blot analysis of vector capsid composition, the capsid protein-specific antibody B1 (Progen) and an horseradish peroxidase (HRP)-coupled secondary antibody (10004302, Cayman chemical) were used as described previously.^35^

### Transduction experiments

HEI-OC1 and SV-k1 cells^87^ were cultured in DMEM (Gibco) supplemented with 1% penicillin, 1% sodium pyruvate, and 10% fetal calf serum (FCS). For experiments, HEI-OC1 or SV-k1 cells were incubated at 2 × 10^4^ cells/well in a 96 well-plate or 1 × 10^5^ cells/well in a 48-well plate format. Transduction efficiency was analyzed 48 hpt by flow cytometry, measuring 10,000 events using a Cytoflex instrument (Beckman Coulter) and FlowJo software (FlowJoTM 10, version 10.2, FlowJo LLC), unless otherwise stated.

### Structural modeling and heparin docking

The protocol was adapted from Meumann and colleagues and executed as previously described.^73^ AAV capsid variants were modeled using AlphaFold2 (https://www.nature.com/articles/s41586-021-03819-2) and the Rosetta software suite for molecular modeling and design^88^ through RosettaScripts.^89^ The structure of AAV2 (Protein DataBank ID: 6IH9^51^) was relaxed^90^ and used as a reference for all subsequent studies. For heparin docking studies, the GlycanDock^91^ protocol was employed. For the full protocol, including input XML files, see the supplemental information. Figures were created with ChimeraX.^92^

### Heparin affinity and competition assay

Heparin affinity chromatography was performed as previously described.^73^^,^^74^ Samples were analyzed by qPCR on a LightCycler 96 real-time PCR system (Roche) using eGFP primers.

For the competition assay, HEI-OC1 cells were seeded in 96-well plates. scAAV.CMV-eGFP vectors (GOI 500) were incubated with the indicated heparin concentrations in DMEM/1% penicillin/1% sodium pyruvate/10% FCS for 30 min at room temperature. Cells were then incubated with the AAV/DMEM/heparin solution for 48 h, followed by flow cytometry.^73^

### *In vivo* delivery

Mice were anesthetized with an intraperitoneally administered mixture of ketamine (100 mg/kg), xylazine (5 mg/kg), and acepromazine (2 mg/kg). The PSCC was exposed as previously described.^93^ In brief, after applying eye protection and positioning the mouse on a heating pad, the animal was prepped with betadine. A 5 mm postauricular incision was made on the left side. The cartilaginous ear canal was traced to the junction with the bony canal, and the facial nerve was identified and preserved. Soft tissue was dissected posterior-superiorly until the posterior canal was identified. The canal was opened with a sterile 18-gauge needle, and the opening was covered with hyaluronic acid gel (Healon, Abbott Labs, Chicago). Vector injections consisted of vector in a 1 μL volume, adjusted to deliver the indicated amounts of vector genomes (vector dose), delivered via a 2.5 μL Hamilton 600 syringe (Part 7632-01) mounted on a micromanipulator. The canalostomy was sealed with a small muscle plug, and the wound was closed with absorbable sutures. The animal was recovered on a heating pad.

### Uncoating assay

For *in vitro* uncoating, HEI-OC1 cells treated with AAV vectors were harvested at the indicated time points, washed with PBS, extensively treated with trypsin, and washed again. Cell pellets underwent subcellular fractionation (Thermo Fischer Scientific, # 78840), and DNA from the nuclear fraction was extracted (DNeasy Tissue kit, Qiagen) and eluted in 30 μL. For each sample, 15 μL of DNA was treated with T5 exonuclease (Biolabs, M0363, 30 Unit) at 37°C for 30 min and diluted 1:2 to inactivate the enzyme. In parallel, 15 μL of the same DNA sample was mock-treated. Quantification of vector genomes was performed by qPCR using transgene-specific primers, with mouse horseradish peroxidase (*hprt*) used as a reference. The percentage of episomal DNA was calculated from the ratio of T5-resistant transgene DNA to total transgene DNA after normalization to non-transduced controls.

For *in vivo* uncoating, anesthetized 1-month-old C57BL/6 mice were injected with 3E8 vg of AAV2 or AAV.MPI into the PSCC. Mice were sacrificed after 1, 3, or 7 days, and the treated cochleae were isolated for DNA extraction of whole cochleae (DNeasy Tissue kit, Qiagen). T5 exonuclease treatment was performed as previously described, with the modification that 1 μg DNA from each sample was either T5-treated or mock-treated.

Immunofluorescence-fluorescence in situ hybridization (IF-FISH) analysis was performed as described previously.^48^

### Capsid thermal stability assay

A thermal stability assay was performed as previously described.^35^

### *In vivo* biodistribution assay

Anesthetized 1-month-old C57BL/6 mice were injected with 3E8 vg of AAV2-CMV-dTomato or AAV.MPI-CMV-dTomato into the PSCC as described above. Mice were sacrificed after 7 days, and brain, liver, injected cochleae, and contralateral cochleae were isolated for subsequent DNA extraction (DNeasy Tissue kit, Qiagen) from 20–30 mg of tissue. DNA (20 ng) was analyzed by qPCR on a LightCycler 96 real-time PCR system (Roche) using CMV primers and mouse *hprt* for reference, with amplification stopped after 30 cycles (background level). qPCR products were visualized using 1% agarose gel electrophoresis (GelDocTM XR+, Bio-Rad).

### HC ablation

HC ablation was carried out via a single subcutaneous injection of kanamycin dissolved in PBS (1 mg/g body weight; Sigma-Aldrich), followed 30 min later by an intraperitoneal injection of furosemide (0.4 mg/g body weight; Sigma-Aldrich). To verify the efficacy of the ototoxin treatment, mice were treated as described above and evaluated for hearing by ABR after 2 weeks.

### ABR measurement

ABR thresholds were recorded using the Intelligent Hearing Systems Smart EP program (IHS, Miami, FL, U.S.A.). Animals were anesthetized as described above and kept warm on a heating pad (37°C). Needle electrodes were placed on the vertex (+), behind the left ear (−), and behind the opposite ear (ground). Tone bursts were presented at 4, 8, 16, and 32 kHz, with a duration of 500 μs using a high-frequency transducer. Recordings were carried out with a total gain equal to 100K and high and low-pass filters set at 100 Hz and 15 kHz, respectively. A minimum of 128 sweeps were presented at 90 dB SPL. The SPL was decreased in 10 dB steps, and near the threshold level, 5 dB SPL steps were used with up to 1024 presentations at each frequency. Threshold was defined as the SPL at which at least one of the waves could be identified in 2 or more repetitions of the recording.

### Immunohistochemistry

Mice were anesthetized via intraperitoneal injections of phenobarbital (585 mg/kg body weight) and phenytoin sodium (75 mg/kg) (Beuthanasia-D Special, Schering-Plough Animal Health Corp.) and sacrificed by intracardiac perfusion with 4% paraformaldehyde in PBS. The temporal bones were removed, the stapes extracted, and the round window opened. Temporal bones were postfixed overnight in 4% paraformaldehyde in PBS at 4°C. After rinsing in PBS three times for 30 min each, the temporal bones were decalcified in 10% ETDA (ethylenediaminetetracetic acid) for 48 h. The temporal bones were then rinsed in PBS, dehydrated, and embedded in paraffin. Ten-μm sections were cut parallel to the modiolus, mounted on Fisherbrand Superfrost/Plus Microscope Slides (Thermo Fisher Scientific), and dried overnight. Samples were deparaffinized and rehydrated in PBS twice for 5 min each, followed by three washes in 0.2% Triton X-100 in PBS for 5 min each, and finally incubated in blocking solution (0.2% Triton X-100 in PBS with 10% fetal bovine serum [FBS]) for 30 min at RT. After blocking, specimens were treated with antibodies listed in Table S1 and diluted to the listed concentration with blocking solution. The tissue was incubated for 48 h at 4°C in a humid chamber. After three rinses in 0.2% Triton X-100 in PBS, immunohistochemical detection was carried out with an Alexa Fluor donkey 488 anti-rabbit (1:1000; Abcam) or Alexa Fluor donkey 488 anti-mouse (1:1000; Abcam). The secondary antibody was incubated for 6 h at room temperature in a humid chamber. The slides were rinsed in 0.2% Triton X-100 in PBS three times for 5 min and finally cover slipped with ProLong Gold antifade reagent (Invitrogen Molecular Probes).

Neuronal survival was quantified in the basal and apical turns of 3 specimens from each treatment group by determining a density of Tuj1 immunostained cells.

### Confocal imaging

All images were acquired using a CSU-W1 Sora confocal laser scanning microscope (Nikon, Japan) with a 20× objective lens. To enhance signal quality, each image was generated as an average of two scans. For each experiment, the gain was optimized based on the brightest image to prevent saturation, and these settings were consistently applied to all subsequent images. No post-processing was performed. Relative intensity measurements were analyzed using ImageJ (https://imagej.net/ij/).

### Statistical analysis

Statistical analysis was performed as indicated in figure legends using unpaired t-tests, one-way ANOVA with Bonferroni post-hoc test, or two-way ANOVA with Tukey’s multiple comparison test, and data are presented as mean (SD). Significance values are defined as ∗*p* < 0.05, ∗∗*p* < 0.01, and ∗∗∗*p* < 0.001. All statistical analyses were performed in GraphPad Prism 5 (GraphPad Software).

## Data availability

The data presented in this study are available on request from the corresponding author.

## Acknowledgments

The authors thank Prof. Jude Samulski (University of North Carolina at Chapel Hill) for providing pXX6 and Federico Kalinec (House Ear Institute, Los Angeles, CA, USA) for providing the HEI-OC1 and SV-k1 cells. We also thank Dr. J.E. Carette (Stanford University School of Medicine) for providing the AAVR.KO cell line of HeLa and HEK293 and the corresponding parental cell lines. This project was funded in part by the 10.13039/501100000781European Research Council under grant agreement no. 819531 to A.S. and by the MWK Lower Saxony-funded Professorinnenprogramm Niedersachsen to H.B. M.E. and C.S. acknowledge financial support from the 10.13039/501100002347Federal Ministry of Education and Research of Germany and the Sächsische Staatsministerium für Wissenschaft, Kultur und Tourismus through the Center of Excellence for AI research, “Center for Scalable Data Analytics and Artificial Intelligence Dresden/Leipzig” (project identification number: ScaDS.AI). S.O.S. was supported by the Institute of Virology, 10.13039/501100006447University of Zurich. A.W., J.H., and O.K. were funded by the 10.13039/501100001659Deutsche Forschungsgemeinschaft (DFG, 10.13039/501100001659German Research Foundation) under Germany’s Excellence Strategy – EXC 2177/1, Project ID 390895286.

## Author contributions

H.B., H.S., A.S., and A.W. supervised the project. J.M., H.B. H.S., S.O.S., C.S., M.E., designed the experiments, J.M., P.H., H.S., S.O.S., and M.E. conducted the experiments and analyzed the data. J.M., O.K., J.H., and J.W. acquired the data. O.K., J.H., A.W, J.S., and C.T.S. provided technologies or tools. P.N.-J. performed the statistical analysis. J.M. and H.B. wrote the manuscript. H.B. and A.S. provided funding. All authors reviewed and edited the manuscript.

## Declaration of interests

H.B., H.S, A.S., and J.M. are inventors on a patent for AAV gene therapy for the inner ear. H.B. is an inventor on patent applications focusing on AAV capsid engineering.

## Supplemental information

Document S1. Figures S1–S9 and Table S1

Document S2. Article plus supplemental information
