# Supplementary material for: Capsid-engineered AAV vector overcomes a key intracellular barrier and efficiently transduces spiral ganglion neurons in adult mice
Source: Mol Ther Adv. 2026 Jan 13;34(1):201669. doi: 10.1016/j.omta.2026.201669 (PMC13148912; doi:10.1016/j.omta.2026.201669)
Supplement: Document S1. Figures S1–S9 and Table S1 [file mmc1.pdf]

OMTA, Volume *34*

Supplemental information

Capsid-engineered AAV vector overcomes a key intracellular barrier and efficiently transduces spiral ganglion neurons in adult mice

Jennifer Marx, Peixin Huang, Sereina O. Sutter, Moritz Ertelt, Odett Kaiser, Jennifer Harre, Juliane Schott, Josephine Wilkes, Philipp Neek-John, Axel Rossi, Athanasia Warnecke, Clara T. Schoeder, Axel Schambach, Hinrich Staecker, and Hildegard Büning

Supplemental information – Methods

Isolation of mouse inner ear cultures

Postnatal day three C57BL/6 mouse pup temporal bones were removed and the organ of Corti was isolated. The organs were then washed in Dulbecco's modified Eagle's medium (DMEM) and cultured on a Millicell™ membrane (Merck Millipore) suspended in 1,500 µL of DMEM supplemented with N1 (Sigma) plus 100 µL/mL penicillin and 5.5 µL/mL of 30% glucose + 1 mM neomycin and maintained *in vitro* (37 °C, 5% CO_2). After 48 h of neomycin exposure, the explants were exposed to phage display selection screen.

Phage display screen

A phage library (New England Biolabs) displaying random 7-mer peptides was used for panning with mouse inner ear cells (2 x 10^{11} plaque-forming units (pfu) in 200 µL of 1 x PBS buffer) and incubated 4 h at 4 °C with shaking. The cells were washed 10 x with Tris-buffered saline (TBS)/0.1 % Tween under direct visualization and the remaining surface-bound phage eluted for 10 min with 100 µL of 0.2 M glycine-HCl (pH 2.2), and neutralized with 15 µL of 1 M Tris-HCl (pH 9). The phage were amplified in *E.coli*, titered and subjected to three further rounds of panning with 2 x 10^{11} pfu of the amplified phage pool from the previous round added to mouse inner ear cells. All panning experiments were performed in duplicate.

Characterization of phage display library

After four rounds of panning, isolated colonies were streak plated on X-gal/IPTG plates. Individual colonies were used to infect early log phase ER2738 host bacteria and amplified for 4.5 h at 37 °C. The bacteria were pelleted by centrifugation and phage recovered from the supernatant using QIAprep Spin M13 kit (Qiagen) and resuspended in 50 µL molecular grade

water. Single stranded DNA quantity was determined by absorption spectrophotometry and the 7-mer peptide DNA coding regions were sequenced (5'-CCC TCA TAG TTA GCG TAA CG-3').

Identification of phage binding regions in the mouse inner ear

Candidate 7-mer peptide epitopes were selected, and colonies amplified and recovered as previously described. Two months old female C57BL/6 mice were anesthetized with an intraperitoneally administered mixture of ketamine (100 mg/kg body weight), xylazine (5 mg/kg body weight) and acepromazine (2 mg/kg body weight). A dorsal postauricular incision was made and the bulla was exposed and opened. After identifying the round window niche, phages (1 x 10^9 pfu) were injected into the scala tympani using a Hamilton micro syringe (Hamilton Company). The opening was sealed with a piece of tissue. Anaesthesia was maintained and at 1 h post phage inoculation, mice were sacrificed by intracardiac perfusion with 4 % PBS buffered paraformaldehyde. The temporal bones were isolated and decalcified in Calex (Fisher Scientific) for 24 h. The temporal bones were embedded in paraffin and cut in 10 µm sections. Sections were deparaffinized and blocked. Primary antibody directed against the M13 protein (rabbit anti-M13AbD Serotec, 1:1000) was added to tissue samples and allowed to incubate for 12 h at 4 °C. The samples were washed in PBS and incubated with fluorescent secondary antibody (goat anti-rabbit, FITC, 1:50) for 12 h at 4 °C. After washing, tissue sections were visualized and photographed via fluorescent microscopy.

Luciferase activity assay

Two-month-old C57Bl/6 mice were anesthetized and AAV.MPI-CMV-Renilla luciferase at doses 1 x 10^5 , 1 x 10^7 and 1 x 10^9 vector particles (n=3 per group) in 1 µl volume injected into the PSSC as described in methods. A separate cohort, serving as non-vector control, were anesthetized and 1 µl of saline were delivered into the PSSC (n=3). Five days after the injection, mice were

euthanized and the treated cochleae were removed. Cochleae were morselized in the lysis buffer provided in the PierceTM Renilla luciferase Glow Assay kit (Thermo Scientific, Catalog No. 16166). Luciferase assay reactions were prepared according to the manufacturer's instructions. The samples were normalized with same total amount of protein for each reaction, and each cochlea was measured in triplicate. Luciferase activity was quantified using a Tecan Infinite M200PRO luminescence reader.

Statistical analysis

Analyses of luciferase activity assay were performed in R (≥ 4.3) using the tidyverse (*readxl, dplyr, tidyr, purrr)* for data import and manipulation, *pwr* for post hoc power calculations, *effectsize* (via *broom*) for bias corrected Cohen's d (Hedges *g*), and *flextable* for table output. The Excel sheet with measured values was read and the mean of the three technical replicates was computed; vector dose was treated as a factor. For every vector dose, a two sample Welch t test (due to large difference in variance) against the control reference was run, the corresponding Hedges *g* and power were calculated, and all raw p values were adjusted for multiple testing using the Benjamini Hochberg FDR method.

Structural modeling and heparin docking

AAV capsid variants were modeled with the Rosetta software suite for molecular modeling and design[1,2] and AlphaFold2.[3] The structure of AAV2 (PDB ID: 6IH9)[4] was relaxed[5,6] and used as reference for all further studies (Listing 1).

Listing 1: Rosetta relax command

```
~/Rosetta/main/source/bin/relax.linuxgccrelease -s ./6ih9.pdb -ex1 –ex2 –
constrain_relax_to_start_coords
```

Next, we docked a heparin molecule to the native AAV2 VP1 trimer or predicted variants with GlycanDock[7,8] (Listing 2, Listing 3). A total of 1000 decoys were created, and the top 10% scoring models were ranked for lowest interface energy as determined by the InterfaceAnalyzer application.[9,10]

Listing 2: Rosetta glycan dock command

```
~/Rosetta/main/source/bin/rosetta_scripts.default.linuxgccrelease       -
parser:protocol   dock.xml   -s   ./aav2_trimer.pdb   -nstruct   1000   -
include_sugars  -maintain_links  -alternate_3_letter_codes  pdb_sugar  -
docking:partners ABC_D
```

Listing 3: Rosetta XML for glycan docking

```
<ROSETTASCRIPTS>
    <MOVERS>
        <GlycanDockProtocol        name=”dock”        refine_only=”true”
partners=”ABC_D” />
    </MOVERS>
    <PROTOCOLS>
        <Add mover=”dock” />
    </PROTOCOLS>
</ROSETTASCRIPTS>
```

Listing 4: AlphaFold2 prediction

```
run_alphafold.py      --fasta_paths="$1"      --output_dir="./"      --
model_preset=multimer --use_gpu_relax=True --max_template_date=2022-01-01
```

Tables

Table S1: List of primary antibodies used for immunohistochemistry.

Antibody	Company	Product #	Concentration	Dilution	Source	Type
Anti-BDNF	Alomone Labs	ANT-010	0.72 mg/ml	1:50	Rabbit	Polyclonal
Anti-Myosin VIIa	Abcam	ab150386	1.0 mg/ml	1:100	Rabbit	Monoclonal
Anti-Beta-Tubulin III, clone TUJ1	Stemcell Technologies	60052	1.0 mg/ml	1:500	Mouse	Monoclonal

Supplemental information – Figures

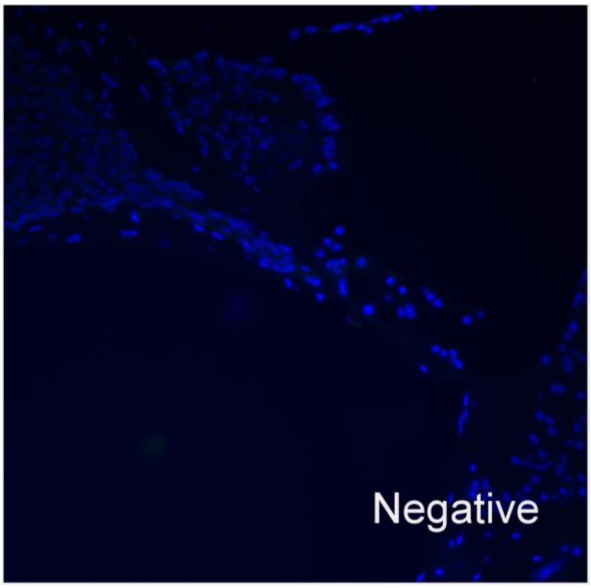

Figure S1: Negative control cochlea shows no dTomato expression. Untreated wild-type mouse cochlea processed in parallel with AAV.MPI-CMV-dTomato–injected samples and isolated 7 dpi as described in the manuscript. No dTomato expression or autofluorescence detected. Blue: DAPI.

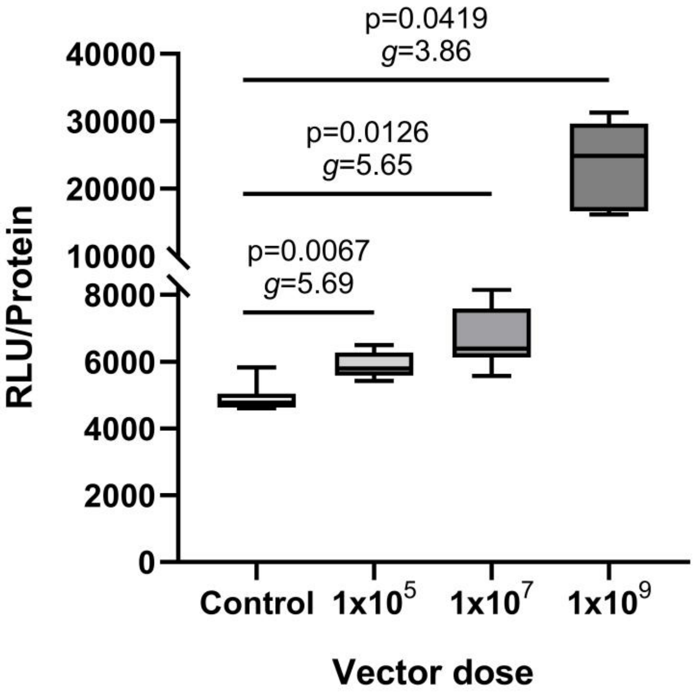

Figure S2: AAV.MPI-mediated expression in cochleae following *in vivo* injection at different doses. Two-month-old C57BL/6J mice were injected via canalostomy with AAV.MPI-CMV-Renilla luciferase vector at doses of 1E9 vg, 1E7 vg and 1E5 vg or saline (control). Cochleae were collected 5 dpi. Renilla luciferase activity in morselized cochleae was measured as relative light units (RLU) using a luciferase assay and normalized to total protein content. n = 3 mice per group. Box and whisker plots show mean with minimum and maximum values. Statistical analysis was performed by Welch's two-sample t-test versus control with Benjamini–Hochberg adjustment for multiple testing. Effect sizes (Hedges *g*) and adjusted p-values are indicated.

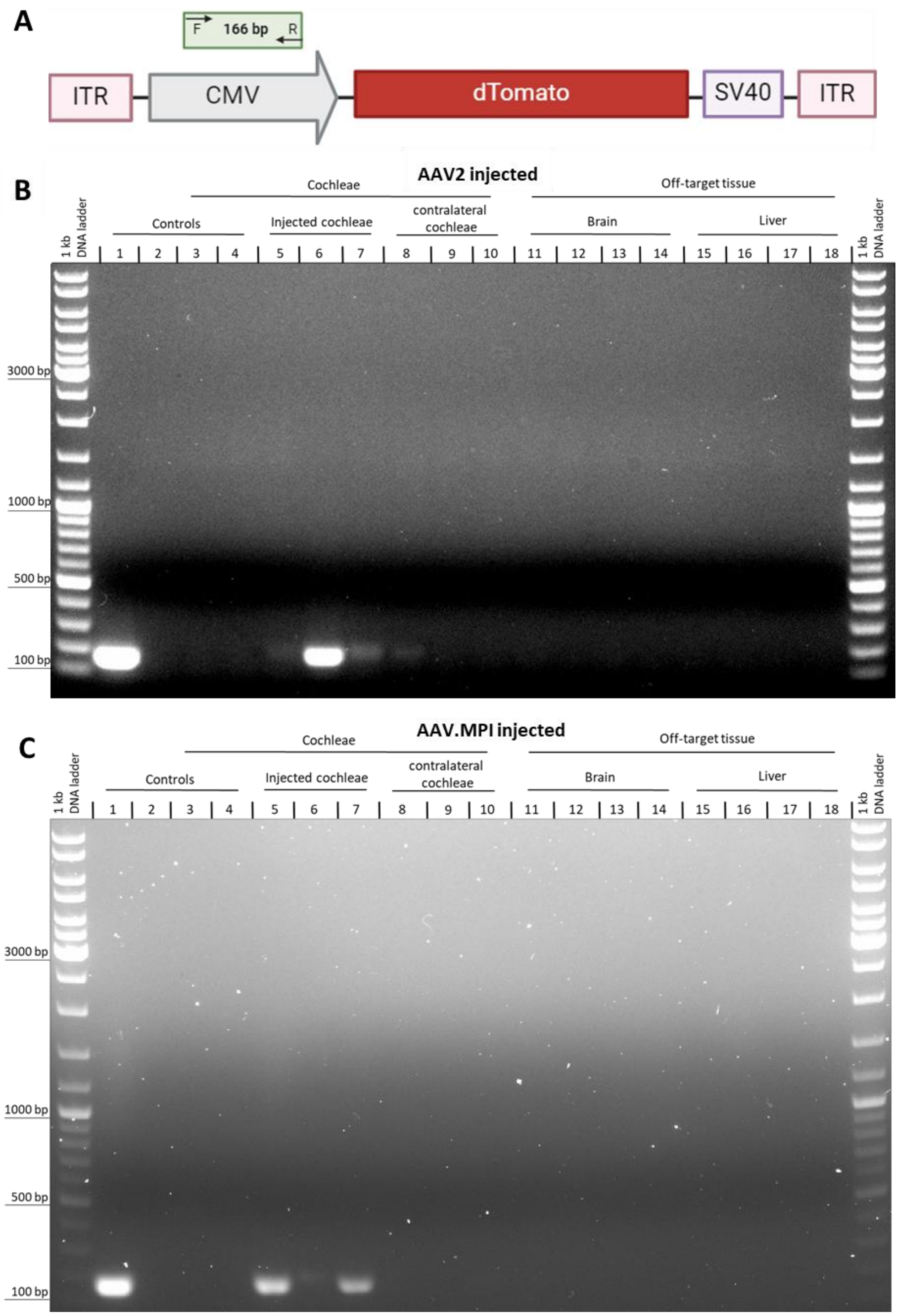

Figure S3: Biodistribution of AAV2 or AAV.MPI injected mice. Mice received either AAV2 or AAV.MPI at 3E8 vg per inner ear trough PSCC injection and were sacrificed after 1 week. **(A)** Schematic representation of AAV vector genome used in the study. Arrows indicate forward (F) and reverse (R) primer. SV40 denotes a polyadenylation signal. **(B-C)** Agarose gel image of amplified PCR products 1: template plasmid DNA, 2: double-distilled H_2O as PCR negative control, 3-4: tissue DNA sample extracted from non-injected left and right cochleae, 5-7:

injected cochlea with either AAV2 **(B)** or AAV.MPI **(C)**, 8-10: tissue DNA samples extracted from contralateral non-injected cochlea, 11: brain tissue DNA sample extracted from non-injected mouse, 12-14: brain tissue DNA sample extracted from injected mouse with either AAV2 **(B)** or AAV.MPI **(C)**, 15: liver tissue DNA sample extracted from non-injected mouse, 12-14: liver tissue DNA sample extracted from injected mouse with either AAV2 **(B)** or AAV.MPI **(C)**. Expected PCR product size is 166 bp. qPCR analysis was terminated at Ct30 at level of background signals.

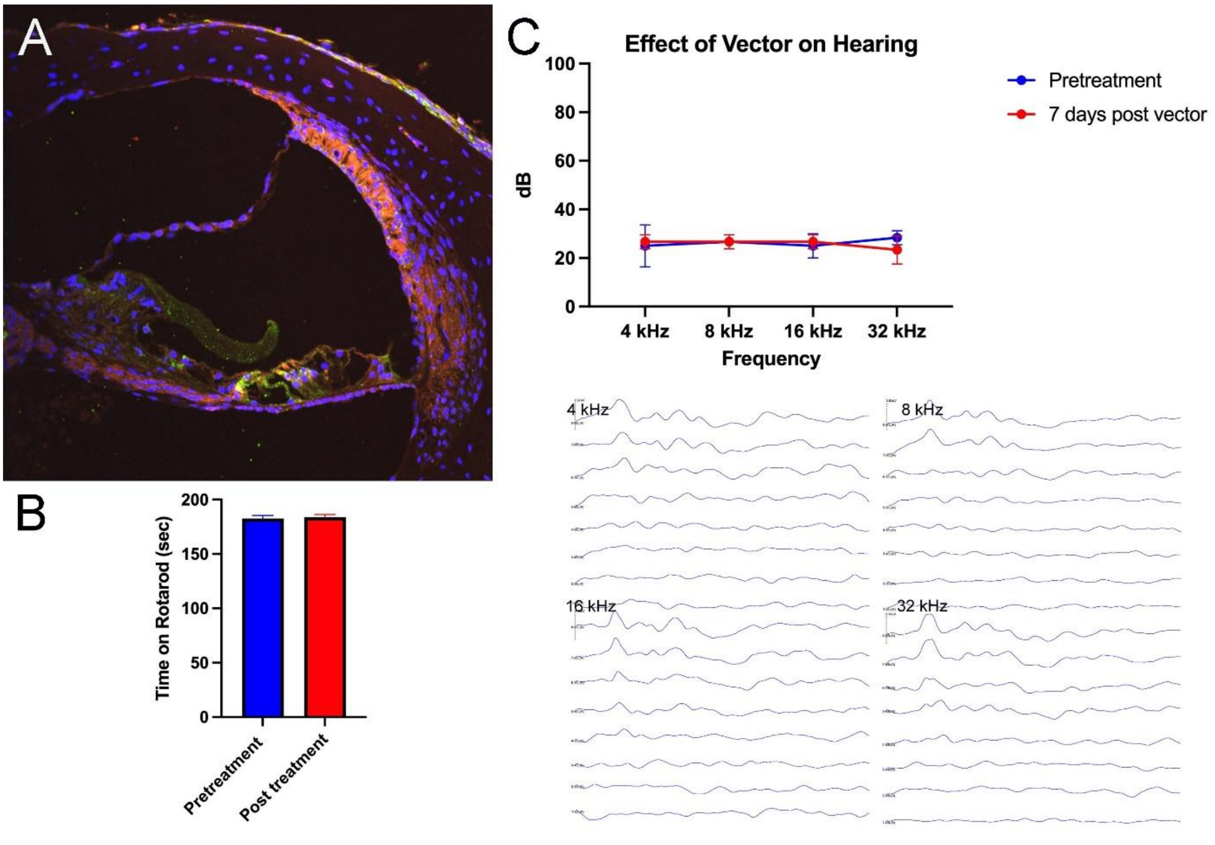

Figure S4: AAV.MPI administration is safe for the inner ear of mice. (A) Immunofluorescent labelling with anti-myosin VIIa (green) in an inner ear of mice treated with dTomato expressing AAV.MPI vector (3E8 vg per inner ear, through PSCC injection, 7 dpi). Hair cells show dual green and red fluorescence and additional dTomato expression can be seen in the neurites and in the lateral wall. **(B-C)** There was no effect of vector delivery in control animals on time on rotarod **(B)** and hearing in ABR measurement **(C)**. Bars and data points represent mean (SD).

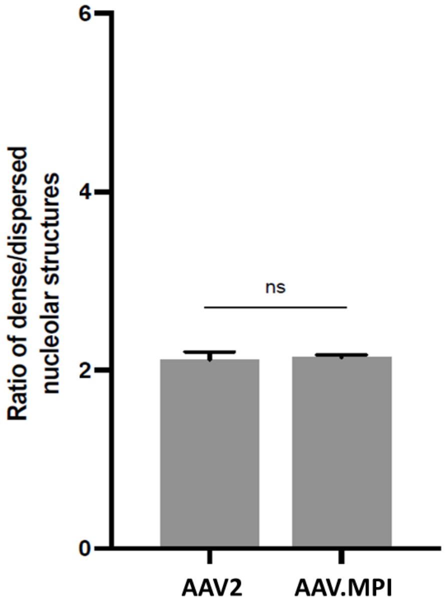

Figure S5: Ratio dense/dispersed nucleoli. NHF cells were infected with AAV2 or AAV.MPI (GOI 20,000) and 24 h later processed for combined IF-FISH, CLSM and image-based quantification of the nucleolar structure of n=50 individual nuclei of AAV2- or AAV.MPI-infected cells. Ratio of dense to dispersed nucleolar structures does not differ between AAV2 or AAV.MPI injected cells. Bars: mean (SD), ns: not significant, unpaired t-test. GOI: genomic particles of infection.

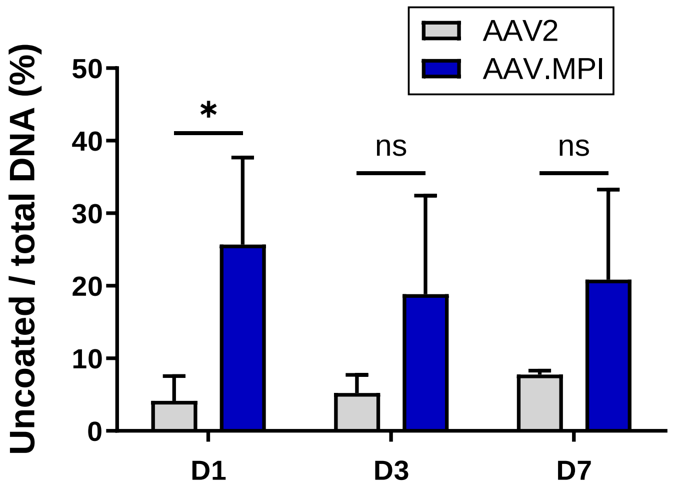

Figure S6: *In vivo* uncoating. One-month-old mice were injected (PSCC) with either AAV2 or AAV.MPI at 3E8 vg per inner ear and were sacrificed after one (D1), three (D3) or seven days (D7). Cochlea were isolated, DNA extracted and indirect uncoating assay performed as described. n = 3 mice for each time point and vector, Bars: mean (SD), ns: not significant, *p<0.05, two-way ANOVA with Bonferroni post-hoc test. vg = vector genome containing particles.

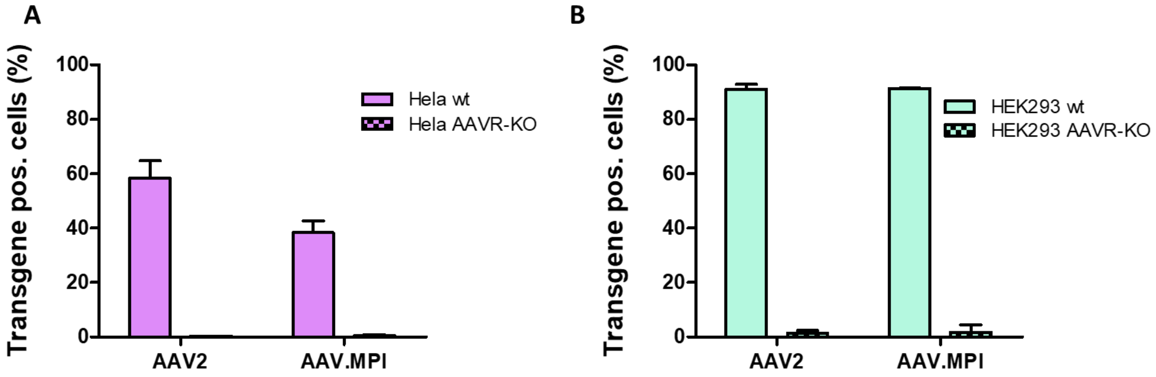

Figure S7: AAVR-KO cell line transduction. Transgene expression of AAV2 or AAV.MPI transduced cells after 24h (GOI 2,500) of **(A)** HeLa wt and HeLa AAVR-KO cells, n = 2 with three technical replicate and **(B)** HEK293 wt and HEK293 AAVR-KO cells, n = 1 with three technical replicates. Bars: mean (SD), GOI: genomic particles of infection.

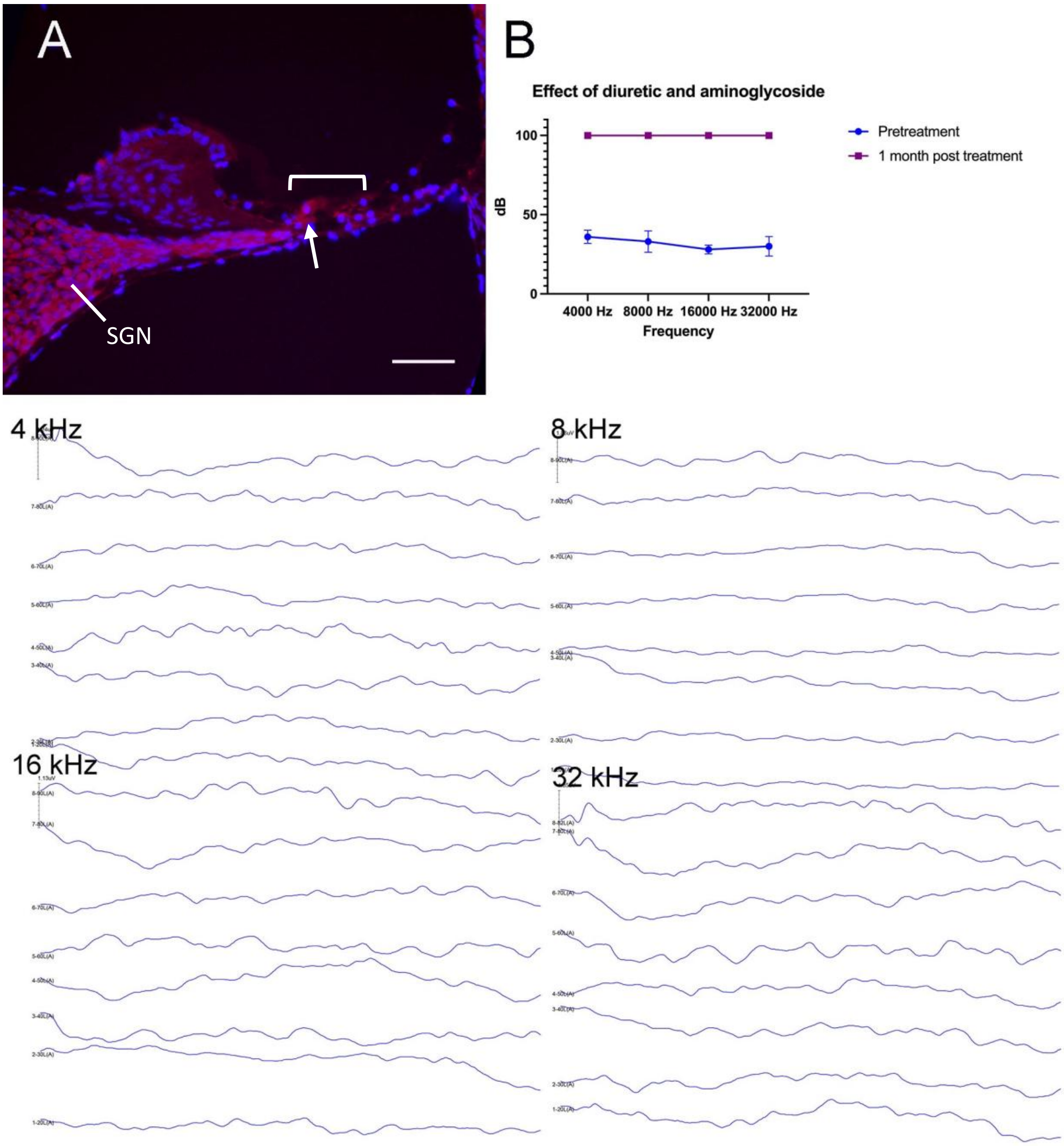

Figure S8: AAV.MPI biodistribution in deafened ear. One month post HC ablation through treatment with aminoglycoside and diuretic, mice were injected with AAV.MPI (3E8 vg per inner ear) through PSCC injection and cochleae isolated 7 dpi. **(A)** AAV.MPI injected cochlea. Bracket: collapse of Organ of Corti with remaining inner phalangeal cell (arrow). dTomato expression in spiral ganglions (SGNs) as seen in healthy mice. Blue: DAPI. **(B)** ABR measurements showing a profound hearing loss within 1 month post treatment with aminoglycoside and diuretic. Scale bar: 50 µm. vg: vector genome containing particles. Data points represent mean (SD).

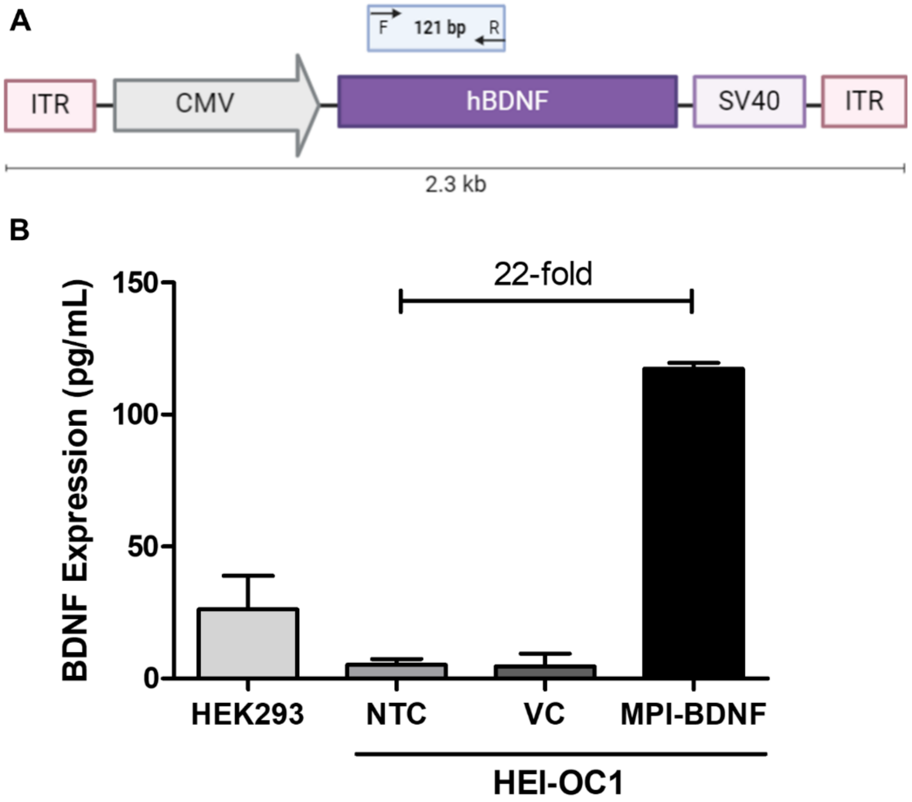

Figure S9: Vector-mediated BDNF expression. (A) Schematic representation of AAV vector genome used in the study with human codon-optimized BDNF (h*BDNF*). Arrows indicate the positions of forward (F) and reverse (R) primers used for vector titration via qPCR. SV40 denotes a polyadenylation signal. **(B)** BDNF expression quantified via ELISA. Supernatant was collected 24h after transduction of HEI-OC1 cells with AAV.MPI-CMV-dTomato (VC, GOI 3,000), AAV.MPI-BDNF (GOI 3,000), and a non-transduced control (NTC). Additionally, supernatant from untreated HEK293 cells was included as a reference control. AAV.MPI-BDNF transduction resulted in a 22-fold increase in BDNF expression compared to non-transduced HEI-OC1 cells, demonstrating the strong expression capability of the vector. GOI: genomic particles of infection. Bars, mean (SD), n=1.

References

1. Leaver-Fay, A., Tyka, M., Lewis, S.M., Lange, O.F., Thompson, J., Jacak, R., Kaufman, K., Renfrew, P.D., Smith, C.A., Sheffler, W., et al. (2011). ROSETTA3: an object-oriented software suite for the simulation and design of macromolecules. Methods Enzymol *487*, 545–574. https://doi.org/10.1016/B978-0-12-381270-4.00019-6.

2. Fleishman, S.J., Leaver-Fay, A., Corn, J.E., Strauch, E.M., Khare, S.D., Koga, N., Ashworth, J., Murphy, P., Richter, F., Lemmon, G., et al. (2011). RosettaScripts: A Scripting Language Interface to the Rosetta Macromolecular Modeling Suite. PLoS One *6*, e20161. https://doi.org/10.1371/JOURNAL.PONE.0020161.

3. Jumper, J., Evans, R., Pritzel, A., Green, T., Figurnov, M., Ronneberger, O., Tunyasuvunakool, K., Bates, R., Žídek, A., Potapenko, A., et al. (2021). Highly accurate protein structure prediction with AlphaFold. Nature 2021 596:7873 *596*, 583–589. https://doi.org/10.1038/s41586-021-03819-2.

4. Zhang, R., Cao, L., Cui, M., Sun, Z., Hu, M., Zhang, R., Stuart, W., Zhao, X., Yang, Z., Li, X., et al. (2019). Adeno-associated virus 2 bound to its cellular receptor AAVR. Nature Microbiology 2019 4:4 *4*, 675–682. https://doi.org/10.1038/s41564-018-0356-7.

5. Khatib, F., Cooper, S., Tyka, M.D., Xu, K., Makedon, I., Popović, Z., Baker, D., and Players, F. (2011). Algorithm discovery by protein folding game players. Proc Natl Acad Sci U S A *108*, 18949–18953. https://doi.org/10.1073/PNAS.1115898108/SUPPL_FILE/PNAS.1115898108_SI.PDF.

6. Tyka, M.D., Keedy, D.A., André, I., Dimaio, F., Song, Y., Richardson, D.C., Richardson, J.S., and Baker, D. (2011). Alternate states of proteins revealed by detailed energy landscape mapping. J Mol Biol *405*, 607–618. https://doi.org/10.1016/J.JMB.2010.11.008.

7. Labonte, J.W., Adolf-Bryfogle, J., Schief, W.R., and Gray, J.J. (2017). Residue-centric modeling and design of saccharide and glycoconjugate structures. J Comput Chem *38*, 276–287. https://doi.org/10.1002/JCC.24679.

8. Nance, M.L., Labonte, J.W., Adolf-Bryfogle, J., and Gray, J.J. (2021). Development and Evaluation of GlycanDock: A Protein-Glycoligand Docking Refinement Algorithm in Rosetta. Journal of Physical Chemistry B *125*, 6807–6820. https://doi.org/10.1021/ACS.JPCB.1C00910/ASSET/IMAGES/LARGE/JP1C00910_0006.JPEG.

9. Lewis, S.M., and Kuhlman, B.A. (2011). Anchored design of protein-protein interfaces. PLoS One *6*. https://doi.org/10.1371/JOURNAL.PONE.0020872.

10. Benjamin Stranges, P., and Kuhlman, B. (2013). A comparison of successful and failed protein interface designs highlights the challenges of designing buried hydrogen bonds. Protein Sci *22*, 74–82. https://doi.org/10.1002/PRO.2187.
